# Repression of sphingosine kinase (SK)-interacting protein (SKIP) in acute myeloid leukemia diminishes SK activity and its re-expression restores SK function

**DOI:** 10.1074/jbc.RA119.010467

**Published:** 2020-03-11

**Authors:** Essam A. Ghazaly, Farideh Miraki-Moud, Paul Smith, Chathunissa Gnanaranjan, Lola Koniali, Adedayo Oke, Marwa H. Saied, Robert Petty, Janet Matthews, Randal Stronge, Simon P. Joel, Bryan D. Young, John Gribben, David C. Taussig

**Affiliations:** ‡ Centre for Haemato-Oncology, Barts Cancer Institute, Queen Mary University of London, London EC1M 6BQ, United Kingdom; § Institute of Cancer Research, Sutton, London, United Kingdom; ¶ Department of Haematology, Royal Marsden Hospital, Sutton, United Kingdom

**Keywords:** Sphingosine kinase (SphK), sphingolipid, ceramide, sphingosine-1-phosphate (S1P), leukemia, lipid metabolism, cell signaling, Acute myeloid leukemia, chemotherapy, cytarabine, hypermethylation, sphingosine kinase interacting protein

## Abstract

Previous studies have shown that sphingosine kinase interacting protein (SKIP) inhibits sphingosine kinase (SK) function in fibroblasts. SK phosphorylates sphingosine producing the potent signaling molecule sphingosine-1-phosphate (S1P). *SKIP* gene (*SPHKAP*) expression is silenced by hypermethylation of its promoter in acute myeloid leukemia (AML). However, why SKIP activity is silenced in primary AML cells is unclear. Here, we investigated the consequences of SKIP down-regulation in AML primary cells and the effects of SKIP re-expression in leukemic cell lines. Using targeted ultra-HPLC-tandem MS (UPLC-MS/MS), we measured sphingolipids (including S1P and ceramides) in AML and control cells. Primary AML cells had significantly lower SK activity and intracellular S1P concentrations than control cells, and *SKIP*-transfected leukemia cell lines exhibited increased SK activity. These findings show that SKIP re-expression enhances SK activity in leukemia cells. Furthermore, other bioactive sphingolipids such as ceramide were also down-regulated in primary AML cells. Of note, SKIP re-expression in leukemia cells increased ceramide levels 2-fold, inactivated the key signaling protein extracellular signal-regulated kinase, and increased apoptosis following serum deprivation or chemotherapy. These results indicate that SKIP down-regulation in AML reduces SK activity and ceramide levels, an effect that ultimately inhibits apoptosis in leukemia cells. The findings of our study contrast with previous results indicating that SKIP inhibits SK function in fibroblasts and therefore challenge the notion that SKIP always inhibits SK activity.

## Introduction

Sphingosine kinase interacting protein (SKIP)^3^ is an anchoring protein that interacts with and inhibits the sphingosine kinases (SKs) (1). The SKs are enzymes that phosphorylate sphingosine (SPH) producing sphingosine-1-phosphate (S1P) a signaling sphingolipid (2). S1P acts via G protein–coupled S1P receptors mediating a number of actions such as enhanced growth (3, 4) and survival (5–7). In contrast to S1P, the sphingolipid precursors of S1P, SPH, and ceramide have anti-proliferative and pro-apoptotic effects (8–11). The relative amounts of S1P and ceramide/SPH in the cell is thought to determine cell fate in what has been termed the “sphingolipid rheostat” (2).

In chronic myeloid leukemia (CML) cell lines, SK/S1P mediated resistance to imatinib (12), whereas increasing ceramide levels via overexpression of ceramide synthase (an enzyme that is responsible for ceramide production) and increased imatinib-induced apoptosis (9). Similarly, SK inhibition resulted in reduced S1P and increased ceramide leading to apoptosis in human leukemia U937 and Jurkat cells (13). S1P antagonists induced apoptosis in leukemic cells through protein phosphatase-2 (PP2A) reactivation and dephosphorylation of cKIT and downstream targets such as pAKT and pERK (14–17). Taken together, these studies suggest a role of SK activity in determining leukemia cell fate and sensitivity to chemotherapy.

One previous report showed that SKIP overexpression inhibited the function of SK in fibroblasts (1). SKIP overexpression reduced activation of extracellular signal-regulated kinase (ERK). ERK is a member of mitogen-activated protein kinase (MAPK) family that is activated by phosphorylation in response to growth factors and producing the active kinase, phospho-ERK (pERK) (18). ERK activation has been linked to cell growth, proliferation, prevention of apoptosis, and drug resistance (19).

Acute myeloid leukemia (AML) is an aggressive malignancy associated with a poor prognosis for many patients. A recent study identified three genes with consistent promoter methylation in AML, sphingosine kinase type 1 interacting protein (*SKIP*), *DPP6*, and *ID4* (20). These three genes were repressed in most AMLs compared with normal tissues as a consequence of promoter methylation. ERK was found to be constitutively overexpressed and activated in AML blasts compared with normal hematopoietic precursors suggesting an important role for pERK in AML (21, 22). Therefore, *SKIP* repression in AML might be expected to increase SK function and S1P levels and increase activation of ERK leading to proliferative and anti-apoptotic effects.

Little is known about SK activity in primary AML cells, nor why the *SKIP* gene is hypermethylated in such a high percentage of AML. The aim of the current study was to investigate the role of *SKIP* repression in AML. We quantified ceramide, SPH, and S1P in primary AML cells using untargeted metabolomic LC–MS (UPLC-MS) and targeted UPLC-MS/MS. This showed down-regulation of all three sphingolipids in AML cells. SK activity was depressed in primary AML cells compared with normal hematopoietic cells. We also developed stable transfections of the *SKIP* gene in myeloid leukemia cell lines. We used these transfections to characterize the metabolic changes associated with *SKIP* re-expression. Consistent with our results in primary AML, *SKIP* transfection enhanced SK function and increased the levels of the three sphingolipids. Our results showed that SKIP is capable of interacting with, and stimulating the function of SK in leukemia cell lines. This was associated with increasing apoptotic signals and chemosensitivity. We conclude that SKIP down-regulation in AML leads to reduced sphingosine kinase activity and reduced ceramide, which ultimately inhibit the apoptosis response.

## Results

### Sphingolipids are deregulated in AML

**Figure 1.**
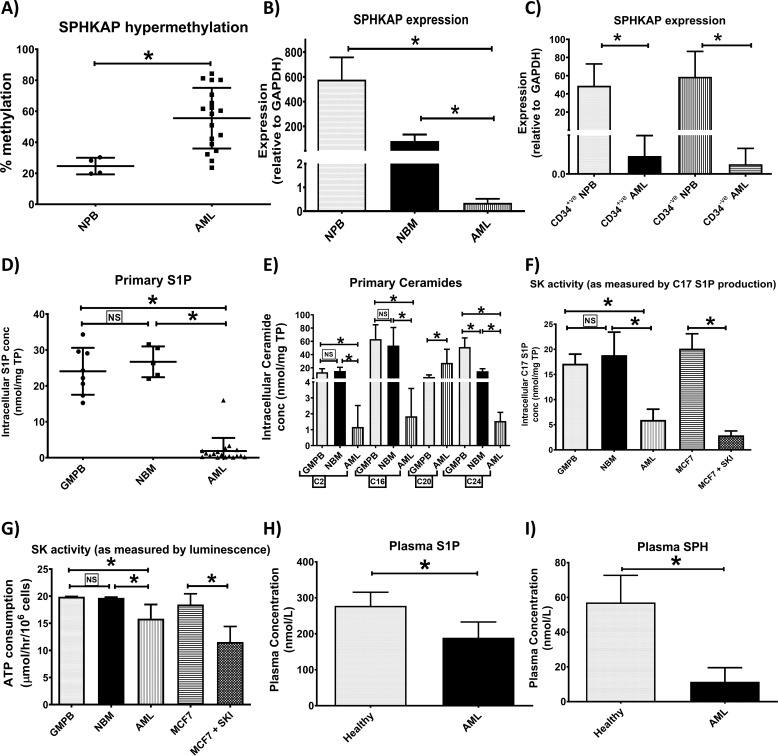
**S1P and ceramides are down-regulated in AML due to SK hypofunction.** Using bisulfite pyrosequencing, *SPHKAP* (the gene that produces SKIP) hypermethylation was confirmed in primary AML (*n* = 18) compared with NPB (*n* = 4) samples (*A*). *SKIP* underexpression was confirmed in blood samples from patients with AML (*n* = 18) compared with healthy volunteer NPB (*n* = 4) and normal bone marrow samples (NBM, *n* = 5) as studied by qPCR (*B*). Reduced *SKIP* expression involved both CD34^+^ and CD34^−^ components of AML primary samples (*n* = 4) compared with NBP (*n* = 4) (*C*) as studied by qPCR. Scatter plots show lower S1P (*D*) and ceramide (*E*) concentrations in primary AML cells (*n* = 18) *versus* NBM (*n* = 5) and G-mobilized peripheral blood cells (GMPB) (*n* = 8). *Bar charts* show lower SK function as measured by UPLC-MS/MS detection of C17 S1P production (*F*) after 24 h incubation with 1 μm C17 sphingosine substrate in primary AML cells (*n* = 6) *versus* NBM (*n* = 5) and GMPB (*n* = 6) MCF7 cell line was used as positive control and 10 μm SKI 5C was used to inhibit SK activity. Lower SK function in primary AML cells (*n* = 18) *versus* NBM (*n* = 3) and GMPB (*n* = 3) was confirmed using another method for measuring SK activity depending on ELISA detection of ATP consumption due to SK enzymatic activity (*G*). Cell lysate from the MCF7 cell line was used as source for SK enzyme (positive control) and 10 μm SKI 5C was used to inhibit SK activity. Plasma S1P (*H*) and sphingosine (*I*) concentrations were lower in AML patients (*n* = 15) *versus* healthy volunteers (*n* = 5) as measured by UPLC-MS/MS. * = *p* < 0.05; *NS*, not significant (*p* > 0.05) as measured by *t* test.

Sphingosine kinase anchoring protein (*SPHKAP*), the gene that produces the SKIP protein was shown to be hypermethylated in primary AML (*n* = 18) compared with normal peripheral blood (NPB, *n* = 4) samples (Fig. 1*A*). Mean percentage hypermethylation was 2.3-fold higher in AML compared with NPB leading to reduced *SKIP* expression in AML (*n* = 18) compared with NPB (*n* = 4) and normal bone marrow (NBM) (*n* = 5) (Fig. 1*B*). *SKIP* was under-expressed in sorted CD34^+^ and CD34^−^ fractions of AML primary samples (*n* = 4) compared with NPB (*n* = 4) (Fig. 1*C*).

Sphingolipids were quantified in primary AML cells using targeted UPLC-MS/MS. S1P intracellular concentrations were reduced in primary AML cells (*n* = 18) compared with NBM and granulocyte colony-stimulating factor mobilized peripheral blood (GMPB) (*n* = 8) used as normal controls (Fig. 1, *D* and *E*). The total cumulative concentration of ceramides C2, C14, C16, C18, C20, and C24 was significantly lower in AML cells than either of the normal controls (*p* < 0.0001, unpaired *t* test). The total cumulative intracellular concentration of ceramides C2, C14, C16, C18, C20, and C24 in AML (*n* = 18) was 20 ± 7.8 nmol/mg of total protein, NBM (*n* = 5) was 83.7 ± 26.8 nmol/mg total of protein, and GMPB (*n* = 8) was 131.8 ± 20.5 nmol/mg of total protein. Ceramides C14 and C18 were undetectable in any of the cells with a lower limit of detection of 290 pmol/liter. The data for ceramide C2, C16, C20, and C24 are shown in Fig. 1*E*. We did not have an appropriate internal standard for C22 and C26 ceramides to allow calculation of a concentration using the targeted approach. There was no significant correlation (data not shown) seen between both intracellular S1P or C2 ceramide and the AML risk group (Table S1).

SK function was determined in primary AML, NBM, and GMPB cells by quantifying C17 S1P using targeted UPLC-MS/MS in cells incubated with its precursor C17 SPH for 24 h. C17 SPH is an uncommon sphingosine in human cells. C17 SPH is converted to C17 S1P by SK in the cells, thus the amount of C17 S1P is a measure of the functional SK activity. MCF7 cells were used as a positive control and the SK1 inhibitor (SKI), 5C, was used with MCF7 cells as a negative control. SK function was significantly down-regulated in AML as indicated by the lower production of C17 S1P compared with NBM and GMPB (Fig. 1*F*).

SK function was also measured by bioluminescence assay. This assay measures remaining ATP following the sphingosine kinase reaction whereby ATP is utilized by sphingosine kinase activity in the cell lysate. After 1 h incubation with SK substrate SPH, ATP consumption was significantly higher in NBM and GMPB cells than AML primary cells (Fig. 1*G*). MCF7 cells were used as a positive control, and together with the SK1 inhibitor 5C as a negative control. This finding confirms reduced SK function in AML primary cells.

Plasma concentrations of S1P were also lower in AML compared with age-matched healthy controls in keeping with the lower SK function in AML patients (Fig. 1*H*). C2 and long chain ceramides were not detectable in many plasma samples and therefore data are not shown (lower limit of detection; 290 pmol/liter). Therefore, we measured SPH plasma concentrations (the product of ceramide and the precursor of S1P) and we found it to be significantly lower in AML compared with healthy controls (Fig. 1*I*). There was no significant correlation between either plasma S1P or SPH and the AML risk group (Table S2).

Changes in SK function could have been related to changes in SK levels. However, we did not detect any significant differences in SK1 or SK2 by either quantitative PCR or Western blotting (Fig. S1). Therefore the reduced SK activity we have observed in AML cells is not due to changes in SK levels.

### Characterization of SKIP expressing cell lines

**Figure 2.**
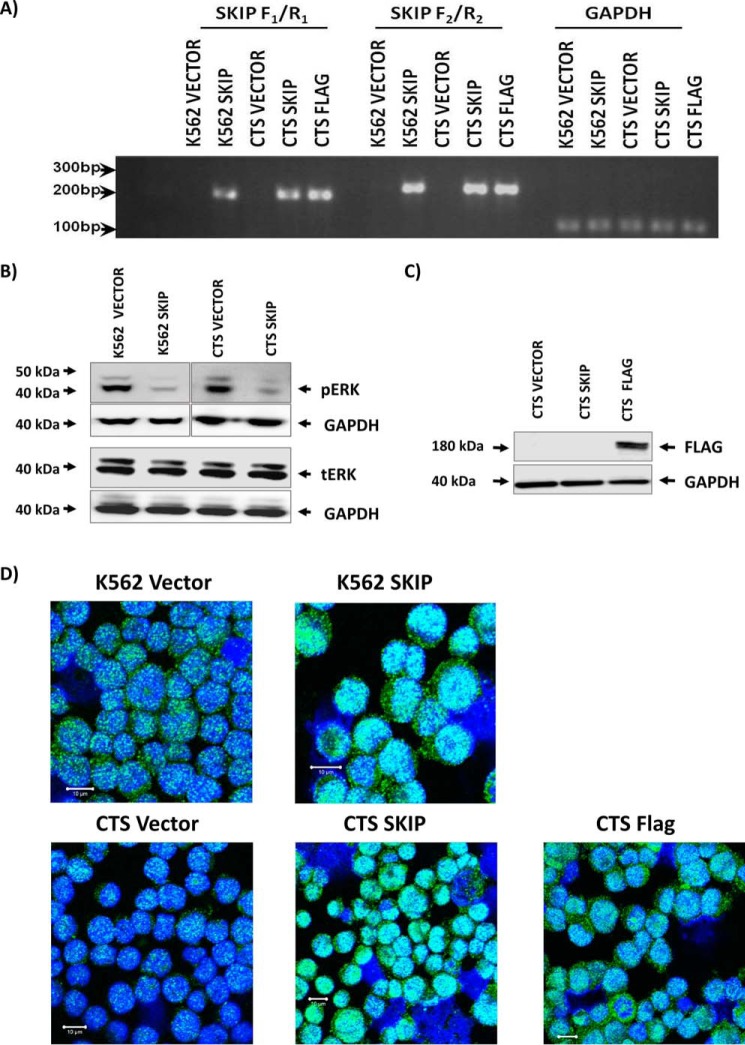
**Successful transfection and re-expression of SKIP protein in K562 and CTS cell lines.** *A, SKIP* gene expression in *SKIP*-transfected cells was confirmed by RT-PCR using two different primers (F_1_/R_1_ and F_2_/R_2_), *GAPDH* expression was used as control for loading. *B,* Western blots show low pERK phenotype associated with SKIP re-expression in K562 and CTS cell lines and equal tERK expression in the two cell lines. *C,* Western blotting confirming the expression of FLAG-tagged SKIP protein in FLAG-tagged transfected CTS cell line. *D,* immunofluorescent detection of SKIP using anti-SKIP antibody and confocal microscopy in SKIP-transfected *versus* vector alone-transfected K562 and CTS cell lines. Fluorescence was detected using a Zeiss LSM 510 META, confocal microscope system. All immunofluorescent images were taken at a magnification of ×40, *scale bar* = 10 μm.

Our results demonstrate down-regulation of SK activity in AML. We therefore sought to establish the relationship between *SKIP* expression and the sphingolipid pathway down-regulation in AML using a transfection model in leukemia cell lines. *SKIP* is silenced by hypermethylation in leukemia cell lines K562 and CTS (20). To study SKIP function, both cell lines were transfected with full-length *SKIP* gene and in addition, CTS cells were transfected with a FLAG-tagged *SKIP* gene. Expression of *SKIP* was confirmed by RT-PCR (Fig. 2). RNA expression was confirmed using two different primer sets (SKIP F1/R1 and SKIP F2/R2). Both primers sets amplified SKIP in transfected cells (K562 SKIP, CTS SKIP, and CTS FLAG) compared with their vector alone transfections (K562 and CTS vector cell lines) (Fig. 2*A*). Neither of the two vector-alone transfected cell lines showed positive expression of either primers confirming that *SKIP* remains normally silenced in these cell lines.

Transient *SKIP* overexpression in fibroblasts has been shown to reduce ERK activation (1). Therefore, pERK expression was tested in K562 and CTS cell lines transfected with SKIP and in FLAG-tagged SKIP transfection into CTS cells. Stable *SKIP* expression resulted in reduced pERK and no change in tERK (Fig. 2*B*). FLAG-tagged SKIP expression was also confirmed in the CTS cell line (Fig. 2*C*). In addition, confocal microscopy confirmed SKIP protein expression in all transfected cell lines (Fig. 2*D* and Fig. S2). SKIP protein expression was increased in *SKIP*-transfected cells, with increases noted in both cytoplasmic and nuclear areas. Taken together, these data demonstrate successful stable transfections in all tested cell lines and confirm the expression of SKIP protein. These cell lines were used in the subsequent experiments to characterize the biological consequences of SKIP re-expression.

### SK activity is increased in SKIP-transfected leukemia cells

**Figure 3.**
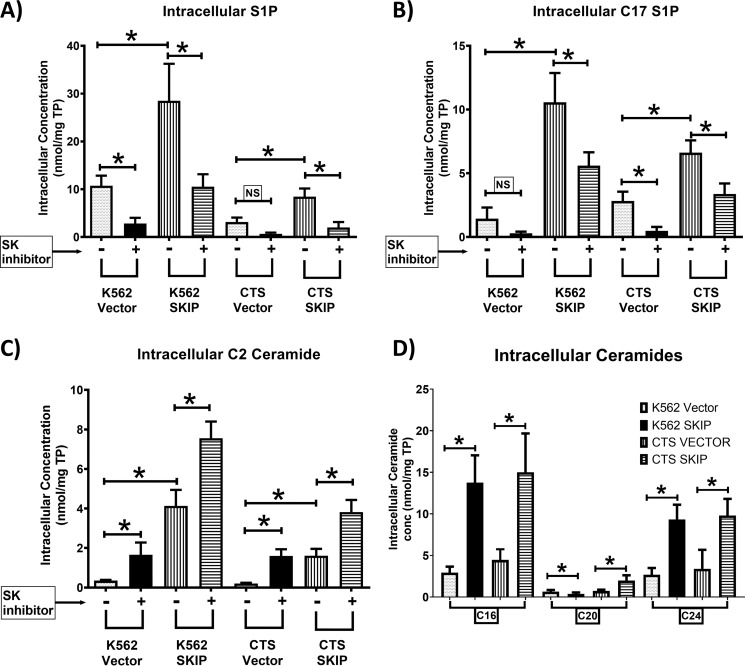
**SKIP overexpression in leukemia cell lines increases S1P and ceramide levels and SK activity.** *A,* higher SIP concentrations in *SKIP*-transfected leukemia cell lines (*n* = 4) compared with vector alone (*n* = 4) as detected by UPLC-MS/MS. The SK1 inhibitor 5C reduced S1P concentrations. *B,* higher SK activity in *SKIP*-transfected (*n* = 4) compared with vector-alone transfected (*n* = 4) leukemia cells as measured by the ability of the cells to produce C17 S1P production after 24 h incubation with C17 sphingosine substrate (*n* = 4). The SK1 inhibitor partially reversed the increased SK activity in *SKIP*-transfected cells. *C,* C2 ceramide was significantly higher in *SKIP*-transfected cells (*n* = 5) and cells cultured with the SK1 inhibitor 5C were compared with vector-alone transfected cells (*n* = 5). *D,* the intracellular long chain (C16, C20, and C24) ceramide levels are shown in *SKIP*-transfected cell lines (*n* = 4) compared with vector-transfected cells (*n* = 4) as detected by UPLC-MS/MS ceramide 14 and C18 were undetectable. * = *p* < 0.05; *NS*, not significant (*p* > 0.05) as measured by *t* test.

We measured S1P concentrations and SK activity in transfected cell lines using targeted UPLC-MS/MS. Intracellular S1P concentrations were higher in *SKIP*-transfected cell lines compared with vector alone (Fig. 3*A*). The increase in S1P in *SKIP*-transfected cells was reversed by the addition of the SK1 inhibitor 5C indicating that the increase in S1P is due to SK1 activity. SK function was increased with a higher accumulation of C17 S1P in *SKIP*-transfected cell lines compared with vector alone (Fig. 3*B*). The addition of the SK1 inhibitor 5C reduced the levels of C17 S1P indicating that the production of C17 S1P depends on SK1 function.

Together, these data indicate an increase in total intracellular SK function and intracellular S1P concentrations associated with *SKIP* gene transfection. These findings strongly support the notion that SKIP enhances SK activity.

SK1 mRNA levels were higher in *SKIP*-transfected cells, whereas the SK2 mRNA levels were lower in *SKIP*-transfected K562 cells (Fig. S3). However, the SK1 and SK2 protein levels as measured by Western blotting were not different in *SKIP*-transfected cells (Fig. S3). Therefore the increased SK activity in the *SKIP*-transfected cells does not appear to be due to a difference in quantity of SK protein present.

### SKIP localizes SK to the cell cytoplasm

**Figure 4.**
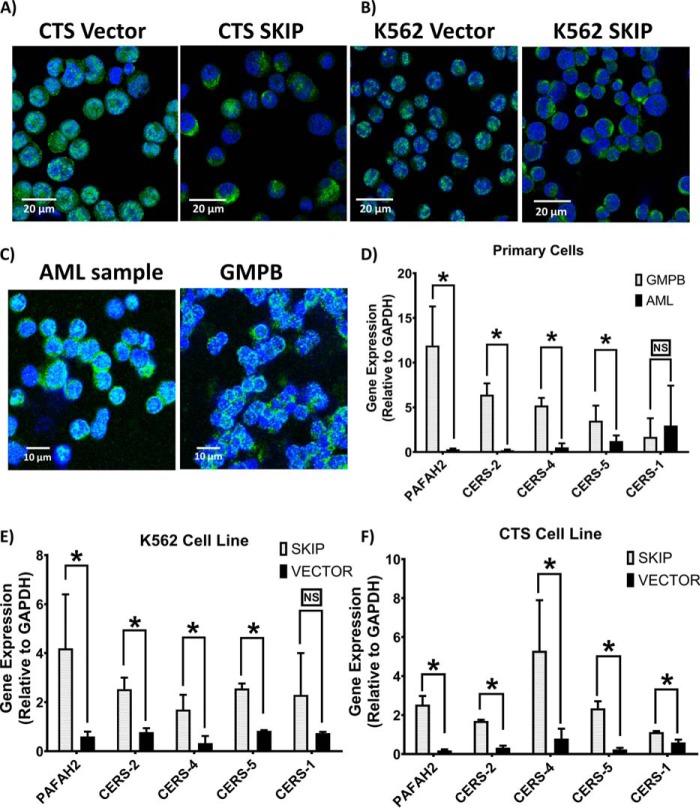
**SKIP protein localizes SK to the cell cytoplasm and increases expression of ceramide synthases in SKIP-transfected cells.** SK1 shows localized expression to cell cytoplasm in *SKIP*-transfected CTS (*A*) and K562 (*B*) *versus* nuclear expression in vector-alone transfected leukemia cell lines. *C,* SK1 expression shows nuclear localization in AML primary cells, whereas localized to the cell cytoplasm in GMPB cells. Fluorescence was detected using a Zeiss LSM 510 META confocal microscope system. All immunofluorescent images were taken at a magnification of ×40 with each *scale bar* = 10 m or 20 μm as indicated in each panel. *D,* lower expression of the genes encoding for ceramide synthases (*CERSs*) and platelet activating factor acetylhydrolase (*PAFAH2*) in primary AML samples (*n* = 11) compared with GMPB (*n* = 6) except for *CERS-1* as determined by RT-qPCR. Lower expression of *PAFAH2* and *CERSs* (except for *CERS-1*) genes in vector-alone transfected (*n* = 3) K562 (*E*) and CTS (*F*) compared with *SKIP*-transfected leukemia cell line (*n* = 3). * = *p* < 0.05; *NS*, not significant (*p* > 0.05) as measured by *t* test.

*SKIP* re-expression caused changes in the expression and distribution of SK in transfected AML cell lines as demonstrated by fluorescence microscopy. MCF7 cells were used as a positive control for SK1 and U266 cells were used as a negative control (Fig. S4, *A–C*). *SKIP* re-expression resulted in redistribution of SK1 enzyme, localizing it to the cell cytoplasm and membrane from the nucleus (Fig. 4, *A* and *B,* and Fig. S4*D*). SK1 expression was found to have nuclear localization in AML primary cells, whereas in GMPB cells SK1 localized to the cell cytoplasm (Fig. 4*C*). These similarities between SK1 expression in GMPB and *SKIP*-transfected leukemia cells confirm the ability of SKIP protein to localize SK1 to the cell cytoplasm. The higher nuclear expression of SK1 in the vector-alone transfected CTS cell line was also confirmed by Western blotting (Fig. S5, *n* = 3). SK2 localization was unaffected by SKIP transfection (Fig. S4*E*).

### Ceramides are increased in SKIP-transfected cells

The intracellular concentration of the ceramides was measured by targeted UPLC-MS/MS in SKIP-transfected cell lines. The total cumulative concentration of ceramides C2, C14, C16, C18, C20 and C24 was significantly higher in *SKIP*-transfected K562 and CTS cell lines ((*p* < 0.0001, unpaired *t* test). The total cumulative concentration of ceramides C2, C14, C16, C18, C20, and C24 was 6.5 ± 0.54 nmol/mg of total protein in vector-transfected K562 cells (*n* = 6) and 26.9 ± 4.2 in *SKIP*-transfected K562 cells (*n* = 6). The total cumulative concentration of ceramides C2, C14, C16, C18, C20, and C24 was 8.8 ± 3.2 nmol/mg of total protein in vector-transfected CTS cells (*n* = 6) and 28.1 ± 6 in *SKIP*-transfected CTS cells (*n* = 6). The values for the individual ceramides are shown in Fig. 3, *C* and *D*. Ceramides C14 and C18 were not detectable in the cell lines. We did not have an appropriate internal standard for C22 and C24 ceramide to allow calculation of the concentration. These results indicate that *SKIP* transfection led to up-regulation in the ceramide synthesis pathway in addition to stimulating SK function.

### Untargeted analysis confirms increased S1P and ceramide levels following SKIP transfection

We then used an *un*targeted UPLC-MS approach to quantify intracellular sphingolipids in *SKIP*-transfected cell lines to provide ancillary evidence for our findings from the targeted UPLC-MS/MS. The identified metabolites and their peak areas were then analyzed using the MetaboAnalyst online tool (SCR_015539). *SKIP* transfection resulted in profound metabolic changes as evidenced by clear separation using principle component analysis of *SKIP* transfected and vector alone cells. The metabolic changes were clearly seen in both intracellular and extracellular (culture medium) compartments (Fig. S6, *A–H*). A consistent up-regulation of S1P, SPH, and ceramide species was observed inside *SKIP*-transfected cells (*n* = 5) and in culture media after 72 h of culture (Fig. S6, *A–H*).

As mentioned above we were unable to quantify the concentration of C22 and C26 ceramide using the targeted approach given a lack of an appropriate internal standard. However, using the untargeted approach we could determine relative amounts of C22 and C26 from the chromatographic area under the curve. C22 ceramide was expressed at a significantly higher level in *SKIP*-transfected cells than vector-transfected cells and in control GMPB compared with primary AML (Fig. S6, *I* and *J*). C26 ceramide was significantly higher in *SKIP*-transfected K562 cells than vector-transfected cells and in control GMPB compared with primary AML (Fig. S6, *K* and *L*). There was no significant difference between *SKIP*-transfected CTS cells and vector-transfected CTS cells. The results from both targeted and untargeted approaches indicate that SKIP is not just impacting S1P production but also a range of sphingolipids including bioactive molecules such as ceramide.

### SKIP transfection up-regulates ceramide synthases

Although SKIP co-localization with SK in the cell cytoplasm can explain the enhanced SK activity and increased intracellular S1P associated with SKIP expression, it does not appear to explain the increases in C2 ceramide and other longer chain ceramides. C2 ceramide can be generated by the activity of platelet activating factor acetylhydrolase (PAFAH2) (23) and longer chain ceramides can be synthesized by activity of different ceramide synthases (CERS) (24). Thus, we studied the expression of ceramide synthase genes (*CERS-1, -2 -4* and *-5*) and *PAFAH2* in primary AML samples and transfected leukemia cell lines. All the genes were found to be significantly under-expressed in AML primary cells compared with GMPB except for *CERS-1* (Fig. 4*D*). Consistent with this, *SKIP* transfection resulted in up-regulation of expression of the same group of genes (again with the exception of *CERS-1*) in transfected leukemia cell lines (Fig. 4, *E* and *F*) compared with vector alone. This confirms the strong association between SKIP expression and the expression of genes involved in ceramide synthesis. Other mechanisms of ceramide production may, however, explain the increase in ceramide levels we observed; the increase appeared to be independent of SK1 activity as C2 ceramide increased further in *SKIP*-transfected cells treated with the SK1 inhibitor 5C (Fig. 3*C*).

### SKIP re-expression leads to increased susceptibility to apoptosis in leukemia cell lines

**Figure 5.**
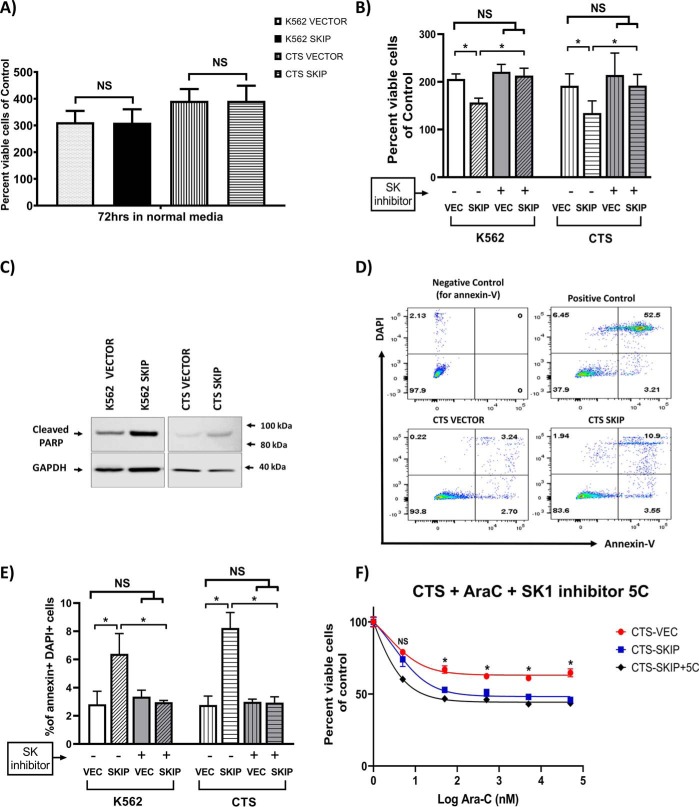
**SKIP re-expression is associated with increased apoptotic signals.** *A, SKIP* transfection did not affect growth of CTS and K562 cells under normal conditions. *B,* after 24 h serum starvation there were significantly less viable *SKIP*-transfected cells than vector-transfected cells for both K562 and CTS cells (*n* = 3). The SK1 inhibitor 5C reversed the pro-apoptotic effect of serum starvation in *SKIP*-transfected cells. *C,* Western blots showing higher expression of cleaved PARP (89-kDa fragment) in *SKIP*-transfected leukemia cell lines compared with vector-alone after 24 h of serum starvation. *D,* flow cytometry plots showing staining of cells with DAPI and Annexin-V (Annexin-V binds apoptotic cells). In the *top left panel* is the negative control for Annexin V. In the *top right panel* is the positive control (irradiated cells) with a high proportion of apoptotic cells (52%). In the *bottom panels* are vector- and *SKIP*-transfected CTS cells showing 3.2 and 10.9% apoptotic cells, respectively. *E,* a higher percentage of cells were apoptotic (positive for both Annexin V and DAPI stains) in *SKIP*-transfected leukemia cell lines exposed to 24 h of serum starvation compared with vector-alone cells (*n* = 3). The proapoptotic effect of *SKIP* transfection was reversed by the SK1 inhibitor 5C in the context of serum starvation. *F, SKIP*-transfected CTS cells were more sensitive to ara-c chemotherapy than vector-transfected cells (*n* = 3). The SK1 inhibitor 5C did not reverse the chemosensitivity to ara-c. * = *p* < 0.05 as measured by unpaired *t test.*

The effect of *SKIP* re-expression on the apoptotic process in leukemia cells was studied. *SKIP* gene transfection did not affect the growth of both cell lines under standard cell culture conditions (Fig. 5*A*). However, SKIP was found to sensitize K562 and CTS cells to the pro-apoptotic effect of 24 h serum starvation. The number of viable cells after culture was assessed using the ViCELL cell viability analyzer (based on trypan blue exclusion). *SKIP*-transfected cells exposed to 24 h of serum starvation showed a significant reduction (*p* < 0.05) in percent viable cell numbers compared with vector-alone cells (Figs. 5*B*).

The expression of apoptotic markers was also studied after 24 h of serum starvation. Cleaved PARP (cleaved by caspases; mainly caspase 3 and 7), 89-kDa fragment concentrations, were higher in *SKIP*-transfected cells compared with vector alone (Fig. 5*C*). This indicates higher caspase and pro-apoptotic activity in *SKIP*-transfected cells in response to serum starvation.

The number of apoptotic cells after 24 h serum starvation was also measured by the Annexin-V flow cytometric assay. Cells staining with both Annexin-V and 4,6-diamidino-2-phenylindole (DAPI) were deemed to be apoptotic (right upper quadrant of each plot in Fig. 5*C*). Apoptosis was significantly higher in *SKIP*-transfected cell lines (Fig. 5, *D* and *E*). These data confirm increased sensitivity to apoptosis in *SKIP*-transfected leukemia cell lines.

The pro-apoptotic effect of serum starvation in *SKIP*-transfected cells was reversed by the addition of the SK1 inhibitor, 5C. This suggests that SK1 mediates the action of SKIP on apoptosis in the context of serum starvation (Fig. 5*B*).

The effect of *SKIP* transfection on sensitivity to chemotherapy was also studied. *SKIP* transfection was found to sensitize transfected cells to the effect of chemotherapy. There were significantly fewer viable cells in the *SKIP*-transfected CTS and K562 cells treated with ara-c chemotherapy, compared with vector-transfected cells (Fig. 5*F*). The SK1 inhibitor 5C did not abrogate the pro-apoptotic effect of ara-c chemotherapy in *SKIP*-transfected cells. This suggests that the pro-apoptotic effects of *SKIP* transfection are not mediated by SK1 in the context of ara-c chemotherapy. The SK1 inhibitor had no effect on the vector-transfected CTS cells (Fig. S7*A*). *SKIP*-transfected K562 cells were also more sensitive to ara-c chemotherapy than vector-transfected cells (Fig. S7*B*). Additionally in K562 cell lines, imatinib had higher cytotoxicity in *SKIP*-transfected cells (Fig. S7*C*). EC_50_ values for imatinib were 2.7 μm/liter (1.2–6.3 μm/liter, 95% confidence interval) in *SKIP*-transfected *versus* 65.1 μm/liter (18.8–225.6 μm/liter, 95% CI) in vector alone K562 cell lines (two-way ANOVA, *p* < 0.0001). Taken together, these data suggest that *SKIP* repression is associated with reduced susceptibility to apoptosis.

## Discussion

Promoter methylation of the *SKIP* gene and associated *SKIP* repression in AML was previously noted by our group (20). We explored the effects of this on SK function using primary AML cells and leukemia cell lines transfected with *SKIP*. We demonstrate that SK function and S1P levels were lower in primary AML cells than controls and that *SKIP* re-expression was associated with an increase in SK activity, and an increase in sphingolipids (mainly S1P and ceramides). Ceramides have a pro-apoptotic effect and this may explain why these changes were accompanied by an increase in apoptotic signals in *SKIP*-transfected leukemia cells and an increase in their chemosensitivity. Inhibition of SK1 did not reverse the enhanced chemosensitivity in *SKIP*-transfected cells consistent with ceramide-mediated apoptosis. In contrast, SK1 inhibition fully reversed the enhanced sensitivity of *SKIP*-transfected cells to serum starvation. These data suggest that serum starvation and chemotherapy are working by different pathways to induce apoptosis.

One previous publication (25) showed increased expression of SK genes by PCR in leukemia, but SK function was not tested. SK requires phosphorylation to function and this may explain the data. The phosphorylation of SK1 mediates translocation of SK1 to the membrane allowing it to function effectively (26). We observed a shift in SK1 distribution in *SKIP*-transfected cells, consistent with this. SK inhibitors do, however, induce apoptosis in cell lines and primary AML cells (13). It is possible that AML cells require some SK function to survive and that inhibiting the already diminished SK activity is fatal for the cell.

SKIP re-expression was associated with an increase in SK function and in S1P intracellular concentrations. This contrasts with data from fibroblast cells in which SKIP expression had a negative effect on SK function (1). However, it should be noted the studies differ in several regards. The *SKIP* transfection methods were different, and our studies were performed in leukemia cells lines, whereas in the previous study the *SKIP* gene was transfected in a nonhematopoietic lineage (fibroblast) cell line. Although we measured the SK activity in intact cells preserving SKIP localization, the previous study measured SK activity in cell lysate, so the localization effect on SK enzyme could not be tested. Critically we also tested the levels in primary cells as well as in patient plasma, with consistent results in all assays. Our data therefore suggest that SKIP functions to support rather than impair SK function, at least in the context of leukemia cells. SKIP may function differently in fibroblasts.

Cellular translocation of proteins through mutation is a recognized event in AML. NPM1 mutation, present in 35% of AML, results in translocation of a nuclear protein to the cytoplasm (27). Here we show translocation of SK from the cytoplasm to the nucleus through epigenetic repression of *SKIP*.

Interestingly, SKIP expression was associated with dephosphorylation and inactivation of pERK. pERK is a prosurvival molecule (19) and is known to be commonly overexpressed in AML (21). Other reports confirmed the ability of C2 ceramide to dephosphorylate pERK as described before (28). Therefore, targeting the MAPK/ERK pathway (22, 29, 30) has been suggested to control proliferation and induce apoptosis in AML blasts. Our results suggest that ERK phosphorylation could be caused by the low concentrations of C2 ceramide associated with SKIP hypermethylation in AML. In our study, *SKIP* re-expression in leukemia cell lines was successful in increasing intracellular C2 ceramide leading to ERK dephosphorylation. This suggests that repairing the SKIP pathway might be a suitable approach to dephosphorylate and inactivate the MAPK/ERK pathway in AML.

We show here that SKIP impacts not just the product of SK, S1P, but also the upstream precursors, SPH and ceramide. This indicates that *SKIP* repression leads to down-regulation of the whole pathway, not just the end product, resulting in lower systemic (*i.e.* plasma) levels of sphingolipids in AML. We show that *SKIP* transfection increases the expression of ceramide synthases and that ceramide synthases are reduced in AML. The lower levels of ceramide in AML are likely to reflect reduced production. Ceramide can also be produced through hydrolysis of sphingomyelin by sphingomyelinases. Sphingomyelinases are mutated in 5% of AML (31), with a number of missense mutations described. Together these data imply that impairment of the ceramide-S1P pathway (either through *SKIP* repression or through sphingomyelinase mutation) is an important process in many AMLs.

Our study is the first study to provide evidence for the suppression of SK activity by *SKIP* methylation in AML. Our data provide convincing evidence that SKIP functions to support SK activity in primary leukemia cells.

## Experimental procedures

### Primary cells and plasma samples

Blood samples and bone marrow were collected from untreated patients with AML after written informed consent at St. Bartholomew's Hospital and the Royal Marsden Hospital. Control bone marrow was obtained from patients with normal blood counts and normal marrow examination who had lymphoma. The protocol was approved by NRES Committee London-City & East (formerly called the East London and The City HA Local Research Ethics Committee 2) on the 31st October 2006 (REC reference 06/Q0604/110) and East of England REC on 14th June 2016 (REC reference 16/EE/0266). All studies comply with the rules of the Review Board and the revised Helsinki protocol. Patient details and characteristics are shown in Tables S1 and S2.

### Primary sample sorting and bisulfite pyrosequencing

Primary samples were incubated for 15 min in phosphate-buffered saline (PBS) with 2% fetal calf serum and CD34 antibody (BD Biosciences, UK). After washing in PBS (+2% fetal calf serum) and DAPI, samples were filtered through a cell strainer and transferred to FACs tubes. Then, samples were sorted using BD FACS Aria II cell sorter (BD Biosciences, UK) into CD34^+^ and CD34^−^ cells. Bisulfite pyrosequencing was done as described (20) using the following primer sequences: forward, GGTTTTATTTAGGGTAGAGTAGATT; reverse (BIOTINYLATED), CCCCCTTCTTTCTATACCCAATACCATATC; sequencing primer, ACCAACTTACCCCAA.

### Cell culture and SKIP transfection method

K562 cells were sourced from the American Type Tissue Collection and CTS cells from Dr. Takeyuki Sato, School of Medicine, Chiba University, Japan. Cells were cultured in RPMI 1640 supplemented with 10% fetal bovine serum, 1% penicillin/streptomycin, and maintained at 37 °C, 5% CO_2_. Cell lines were authenticated using DNA profile (short tandem repeat) analysis and tested for mycoplasma (MycoAlert mycoplama detection kit, Lonza) on a regular basis.

*SKIP* cDNA clone was purchased from Origene and re-cloned into the pIRES Hyg3 expression vector using standard methods. A FLAG tag was added by PCR protocols using High-Fidelity *Taq* polymerase and confirmed by sequencing. Cell lines were transfected with 2 μg of DNA, using the Amaxa Nucleofector according to the manufacturer's instructions. Briefly, 2 × 10^6^ cells were transfected using program T-016, with Solution V. Following transfection, stable cell lines were generated by selection in 200 μg/ml of hygromycin B (Life Technologies, UK). Cell numbers and viability were assessed using the ViCELL cell viability analyzer (Beckman Coulter).

### Cytotoxicity and viability assay

Cells were counted and seeded into 24-well-plates at a concentration of 1 × 10^6^ cells/ml and incubated with 10 μm SKI inhibitor, 5C. Then after 24 h Ara-C added in triplicate at 5 different concentrations. Drug effects were determined after 48 h by counting viable cells using Countbright Absolute Counting Beads (Invitrogen), according to the manufacturer's instructions. The EC_50_ values were calculated by nonlinear regression using an equation for a sigmoidal dose-response curve with variable slope (GraphPad Prism 8.2.1).

### Apoptosis analysis using Annexin V

Cells (1 × 10^6^ cells/ml) were washed using Annexin V-binding buffer (BD Biosciences) and incubated with Annexin V Alexa Fluor 647, for 15 min at room temperature in the dark. Then, cells were washed and re-suspended in PBS with 2% fetal bovine serum and DAPI and analyzed on a BD LSRFortessa cytometer. Dual Annexin V-positive, DAPI-positive cells were defined as apoptotic cells.

### Western blotting

Cell lysates (50 μg) were separated on 4–12% SDS-PAGE and blotted onto polyvinylidene difluoride membranes (Invitrogen, UK). Membranes were incubated with specific antibodies overnight; this was followed by incubation with appropriate secondary antibodies. Then, immunocomplexes were detected by enhanced chemiluminescence (ECL). The primary antibodies used were: anti-phospho-ERK1/2, total ERK, anti-cleaved PARP, anti-GAPDH (New England Biolabs), anti-SK1 (Santa Cruz Biotechnology), anti-SK2 (Abcam), and anti-FLAG (Sigma-Aldrich). Anti-Rabbit or mouse Igs/horseradish peroxidase secondary antibody (Dako, UK) was used. Prostate cells lines were used as a positive control for SK2 (Fig. S8). The cytoplasmic and nuclear fractions of cell lysates were extracted using NE-PER^TM^ Nuclear and Cytoplasmic Extraction Reagents (Thermo Scientific, UK). Quantification analysis of Western blotting bands was performed with ImageJ software.

### RT-qPCR for confirmation of gene expressions

Total RNA was extracted using the RNeasy mini kit (Qiagen, Crawley, UK) according to the manufacturer's protocol and 1 μg of RNA was used for cDNA synthesis via the ImProm-II^TM^ Reverse Transcription System (Promega, Madison, WI). RT-qPCR for SKIP was performed using the Fast SYBR Green Master mix (Applied Biosystems, Warrington, UK). Relative expression of the target gene was determined with respect to housekeeping gene (*GAPDH*) expression. The *SKIP* and *GAPDH* primers were designed in our lab and synthesized by Sigma-Aldrich, UK, as follows: SKIP-F1, CAGTGGATAGCTGCCTCTGA; SKIP-R1, CCCATCCTTCTGCTCCTCAT; SKIP-F2, GTGAGCGCTTGTCAAATCCA; SKIP-R2, CTCATGGATCGCTCACTGAG; GAPDH-F1, CTGCACCACCAACTGCTTAG; and GAPDH-R1, ACAGTCTTCTGGGTGGCAGT. Expression levels of SK1 and SK2 genes were measured using TaqMan real-time PCR assay (Applied Biosystems). Each sample was analyzed in triplicate and normalized to GAPDH (δ*C_T_*). δ*C_T_* values were standardized to a calibrator value generated from the average (δ*C_T_* of all values. The relative expression calculated using the comparative *C_T_* method with fold-change (2^−ΔΔ^*^CT^*).

### Immunofluorescence

Cells were collected, fixed (IC Fixation buffer, 4% paraformaldehyde, from eBioscience), and permeabilized with permeabilization solution (0.1% Saponin and 0.009% sodium azide) before being probed with anti-SPHKAP antibody (Abcam, UK) or anti-SK1 (Santa Cruz Biotechnology). The anti-SPHKAP antibody was validated using positive and negative controls (Fig. S2). After washing and incubation with secondary antibody (anti-rabbit IgG, Alexa Fluor® 488 conjugate, Thermo Fisher Scientific, UK), fluorescence was detected using a Zeiss LSM 510 META, confocal microscope system. U266 cells were used as a negative control for SK1 antibody and these were obtained from ATCC.

### Untargeted UPLC-MS metabolomic analysis

Untargeted UPLC-MS method was used to quantify all intracellular and culture media metabolite contents as described (32). Briefly, cells (5 × 10^6^) were extracted using 80% methanol. Methanolic solution extracts were then evaporated to dryness. Dried extracts were then reconstituted in 50 μl of 10% acetonitrile solution (containing 0.1% formic acid) and only 5 μl was injected into UPLC-MS system. Chromatography was performed on Waters Nanoacquity UPLC system (Waters, UK). Separation of metabolites was achieved on Waters Acquity UPLC HSS T3 column (1.8 μm, 1 × 150 mm) with the following solvent system: A = 0.1% formic acid in water, B = 0.1% formic acid in acetonitrile. The flow rate was 40 μl/min. The analytical run starts by 100% A for 2 min, a gradient of 100 A to 100% B over 10 min, 100% B held for 3 min, then back to 100% A over 1 min. Mass spectrometry was performed on a Waters Q-ToF Premier operated in positive and negative electrospray (ES) ionization modes. ESI voltages were 2.9 (in negative mode) and 3.1 (in positive mode). Cone voltage was 38 V. Source temperature was 80 °C and desolvation temperature was 250 °C. Desolvation and cone gases were nitrogen with flow of 400 and 30 liter/min, respectively. The MS scan was adjusted to acquire between 50 and 1000 *m*/*z* range with scan time of 0.18 s and inter-scan delay of 0.02 s. Data were acquired in profile mode and leucine enkephalin (*M*_r_ = 555.6) was used as Lock mass. Instrument calibration was done by 50 mm sodium formate. A quality control of cell extracts spiked with metabolite mix was injected regularly to monitor the stability of the UPLC system. Data acquisition was done using Water Masslynx software (V 4.1) from Waters, UK. Continuous lockmass correction was done using Masslynx software. Mass detection, chromatographic peak detection, peak deconvolution, deisotoping, retention time normalization, and peak alignment were all done using MZmine software (version 2.2) (36). Peak lists including retention, *m*/*z*, and peak intensities were exported from MZmine and imported into Microsoft Office Excel 2007 (12.0.6214.1000), fold-change and *p* value were then calculated and volcano plots were generated.

### Targeted UPLC-MS/MS analysis of intracellular and plasma S1P, SPH, C17 S1P, C17 SPH, C2 ceramide, and long chain ceramides

The extraction method was modified from published methods (33). Cells (5 × 10^6^) were extracted using chloroform/methanol solution (1/2, v/v) containing internal standards (C17 SPH and C17 S1P). The single-phase extract was incubated overnight at 48 °C. After cooling, interfering glycerolipids were removed by addition of 1 m KOH solution for 2 h followed by neutralization with glacial acid. The single-phase was then evaporated, dried extracts were reconstituted in 100 μl of 80% ethanol (containing 1% formic acid), and 10 μl were injected into the UPLC-MS/MS system. For plasma samples, 20 μl of plasma was extracted with 75 μl of methanol and 5 μl of internal standard solution (C17 SPH and C17 S1P). After incubation on ice for 30 min and centrifugation at 10,000 × *g*, supernatant was transferred to LC-MS vials and injected into the UPLC-MS/MS system.

Analytes were separated on a Waters Kinetex PFP (Pentafluorophenyl) 1.7-μm column, 2.1 × 50 mm, S1P, C17 S1P, SPH, C17 SPH, and C2 ceramide were separated using gradient elution of mobile phase A (water + 0.1% formic acid) and mobile phase B (acetonitrile + 0.1 formic acid). The gradient starts by 70% A, a gradient to 20% A over 3 min, then 20% B for 1 min, then back to 70% A for the 3 min, all at a flow rate of 250 μl/min. For long chain ceramide analysis (including C17 SPH as an internal standard), a gradient elution of methanol/water/formic acid (61:38:1, v/v) with 5 mm ammonium formate to methanol/acetonitrile/formic acid (39:60:1, v/v) with 5 mm ammonium formate at a flow rate of 250 μl/min was employed following a published method (34).

Analytes were detected using triple-stage-quadrupole MS (TSQ Vantage, Thermo Scientific, UK) equipped with an electrospray ion source. Samples were analyzed in the Multiple Reaction Monitoring (MRM), positive ionization modes at a spray voltage of 3500 V. Nitrogen was used as sheath and auxiliary gas at a flow rate of 40 and 10 arbitrary units, respectively. Argon was used as collision gas with pressure of 1.5 mtorr. The optimum transitional daughter ions mass and collision energy for S1P were: *m*/*z* 380 → 264 (collision energy (CE) 11 V), SPH: 300 → 264 (CE28 V), C17 S1P was: *m*/*z* 366 → 250 (CE 11 V), C17 SPH: *m*/*z* 286 → 250 (CE 28 V) and C2 CER *m*/*z* 342 → 264 (CE 18 V), C14 CER *m*/*z* 510 → 264.0 (CE, 16 V), C16 CER *m*/*z* 524 → 264 (CE, 20 V), C18 CER *m*/*z* 566 → 264 (CE, 18 V), C20 CER *m*/*z* 594 → 264 (CE, 18 V), and C24 CER *m*/*z* 649 → 264 (CE, 20 data acquisition and chromatography analysis was carried out using Xcalibur chromatography software version 2.2 from Thermo Scientific, UK.

The targeted UPLC-MS/MS was validated using internal calibration standards (C17 S1P and C17 SPH) and six external calibration standards (0, 0.1, 0.3, 1, 3, and 10 μm concentrations) of each analyte. A high and low (5 and 0.5 μm concentrations) were injected regularly to monitor the stability of the UPLC-MS/MS system. The calibration curves were all linear and the quality control samples inaccuracy and imprecision were all within ±15%. Representative chromatograms for blank samples, quality control, and analyzed samples are shown in Fig. S9, including the intracellular and plasma sphingolipids and long chain ceramides.

### SK activity assay (UPLC-MS/MS) using C17 sphingoid base

SK activity was measured using C17 sphingoid base as described (35). Exponentially growing cells were grown in 10-ml flasks, cells were grown at a concentration of 1 × 10^6^/ml for 24 h. Cells were then incubated with 1 μg/ml of C17 SPH for another 24 h after which cells were pelleted and extracted using chloroform/methanol (1:2, v/v). Concentrations of C17 S1P in cell extracts were then determined using targeted a UPLC-MS/MS technique (see previous section). The MCF7 cell line was used as a positive control and was obtained from the European Collection of Authenticated Cell Culture. The SKI 5C was obtained from Sigma-Aldrich.

### Sphingosine kinase activity assay (ELISA)

SK activity was measured using Sphingosine Kinase Activity Assay kit from Echelon Biosciences, USA (product number: K-3500) according to the manufacturer's protocol. Briefly, cell lysate was extracted from 4 million cells, using cell lysis buffer, containing 50 mm Tris-HCl, 1 mm EDTA, 150 mm NaCl, 0.1% lauryl sulfate, 0.5% deoxycholic acid, and 1% Igepal CA-630. The experiment was then set up in a white 96-well-plate, with ATP calibration standards ranging from 10 to 0 μm. The standards and the samples supplemented with the reagents provided by the kit (50 μm DTT, 10 mm sphingosine, 10 mm ATP and reaction buffer) were added to the wells appropriately. The plate was then sealed and incubated for 1 h at room temperature, before an ATP detection reagent was added to the wells. The plate luminescence was then analyzed using a Polarstar Optima plate reader (BMGLabtech, UK).

### Statistics

The Student's *t* test and ANOVA were used to assess differences. Error bars are standard deviation.

## Author contributions

E. A. G., P. S., C. G., M. H. S., R. P., J. M., S. P. J., B. D. Y., J. G., and D. C. T. conceptualization; E. A. G., F. M.-M., P. S., C. G., L. K., A. O., M. H. S., R. P., J. M., R. S., S. P. J., B. D. Y., J. G., and D. C. T. data curation; E. A. G., F. M.-M., P. S., C. G., L. K., A. O., M. H. S., R. P., J. M., S. P. J., B. D. Y., J. G., and D. C. T. formal analysis; E. A. G., P. S., M. H. S., J. M., S. P. J., B. D. Y., J. G., and D. C. T. supervision; E. A. G., P. S., M. H. S., J. M., S. P. J., B. D. Y., J. G., and D. C. T. funding acquisition; E. A. G., P. S., M. H. S., J. M., S. P. J., B. D. Y., J. G., and D. C. T. validation; E. A. G., F. M.-M., P. S., C. G., L. K., A. O., M. H. S., R. P., J. M., R. S., S. P. J., B. D. Y., J. G., and D. C. T. investigation; E. A. G., F. M.-M., P. S., C. G., L. K., A. O., M. H. S., R. P., J. M., S. P. J., B. D. Y., J. G., and D. C. T. methodology; E. A. G., P. S., M. H. S., J. G., and D. C. T. writing-original draft; E. A. G., P. S., M. H. S., R. P., J. M., S. P. J., B. D. Y., J. G., and D. C. T. project administration; E. A. G., F. M.-M., P. S., C. G., L. K., A. O., M. H. S., R. P., J. M., S. P. J., B. D. Y., J. G., and D. C. T. writing-review and editing.

## Supplementary Material

Supporting Information

## Footnotes

3 The abbreviations used are:

SKIP: sphingosine kinase interacting protein
SK: sphingosine kinase
SKI: SK inhibitor
S1P: sphingosine 1 phosphate
SPH: sphingosine
AML: acute myeloid leukemia
UPLC-MS/MS: ultra-high-performance liquid chromatography-tandem mass spectrometry
ERK: extracellular signal-regulated kinases
cKIT: stem cell factor receptor/CD117
pAKT: protein kinase B
SPHKAP: sphingosine kinase anchoring protein
DAPI: 4,6-diamidino-2-phenylindole
MAPK: mitogen-activated protein kinase
NPB: normal peripheral blood
NBM: normal bone marrow
ANOVA: analysis of variance
AML: acute myeloid leukemia
CML: chronic myeloid leukemia
PARP: poly(ADP-ribose) polymerase
qPCR: quantitative PCR
GAPDH: glyceraldehyde-3-phosphate dehydrogenase
CE: collision energy.

## References

[1] Lacaná E., Maceyka M., Milstien S., and Spiegel S. (2002) Cloning and characterization of a protein kinase A anchoring protein (AKAP)-related protein that interacts with and regulates sphingosine kinase 1 activity. J. Biol. Chem. 277, 32947–32953 10.1074/jbc.M202841200 12080051

[2] Spiegel S., and Milstien S. (2003) Sphingosine-1-phosphate: an enigmatic signalling lipid. Nat. Rev. Mol. Cell Biol. 4, 397–407 10.1038/nrm1103 12728273

[3] Olivera A., and Spiegel S. (1993) Sphingosine-1-phosphate as second messenger in cell proliferation induced by PDGF and FCS mitogens. Nature 365, 557–560 10.1038/365557a0 8413613

[4] Xia P., Gamble J. R., Wang L., Pitson S. M., Moretti P. A., Wattenberg B. W., D'Andrea R. J., and Vadas M. A. (2000) An oncogenic role of sphingosine kinase. Curr. Biol. 10, 1527–1530 10.1016/S0960-9822(00)00834-4 11114522

[5] Morita Y., Perez G. I., Paris F., Miranda S. R., Ehleiter D., Haimovitz-Friedman A., Fuks Z., Xie Z., Reed J. C., Schuchman E. H., Kolesnick R. N., and Tilly J. L. (2000) Oocyte apoptosis is suppressed by disruption of the acid sphingomyelinase gene or by sphingosine-1-phosphate therapy. Nat. Med. 6, 1109–1114 10.1038/80442 11017141

[6] Kleuser B., Cuvillier O., and Spiegel S. (1998) 1α,25-dihydroxyvitamin D3 inhibits programmed cell death in HL-60 cells by activation of sphingosine kinase. Cancer Res. 58, 1817–1824 9581819

[7] Pyne N. J., and Pyne S. (2010) Sphingosine 1-phosphate and cancer. Nat. Rev. Cancer 10, 489–503 10.1038/nrc2875 20555359

[8] Nava V. E., Cuvillier O., Edsall L. C., Kimura K., Milstien S., Gelmann E. P., and Spiegel S. (2000) Sphingosine enhances apoptosis of radiation-resistant prostate cancer cells. Cancer Res. 60, 4468–4474 10969794

[9] Baran Y., Salas A., Senkal C. E., Gunduz U., Bielawski J., Obeid L. M., and Ogretmen B. (2007) Alterations of ceramide/sphingosine 1-phosphate rheostat involved in the regulation of resistance to imatinib-induced apoptosis in K562 human chronic myeloid leukemia cells. J. Biol. Chem. 282, 10922–10934 10.1074/jbc.M610157200 17303574

[10] Cuvillier O., Pirianov G., Kleuser B., Vanek P. G., Coso O. A., Gutkind S., and Spiegel S. (1996) Suppression of ceramide-mediated programmed cell death by sphingosine-1-phosphate. Nature 381, 800–803 10.1038/381800a0 8657285

[11] Morad S. A., and Cabot M. C. (2013) Ceramide-orchestrated signalling in cancer cells. Nat. Rev. Cancer 13, 51–65 10.1038/nrc3398 23235911

[12] Salas A., Ponnusamy S., Senkal C. E., Meyers-Needham M., Selvam S. P., Saddoughi S. A., Apohan E., Sentelle R. D., Smith C., Gault C. R., Obeid L. M., El-Shewy H. M., Oaks J., Santhanam R., Marcucci G., et al (2011) Sphingosine kinase-1 and sphingosine 1-phosphate receptor 2 mediate Bcr-Abl1 stability and drug resistance by modulation of protein phosphatase 2A. Blood 117, 5941–5952 10.1182/blood-2010-08-300772 21527515 PMC3112039

[13] Paugh S. W., Paugh B. S., Rahmani M., Kapitonov D., Almenara J. A., Kordula T., Milstien S., Adams J. K., Zipkin R. E., Grant S., and Spiegel S. (2008) A selective sphingosine kinase 1 inhibitor integrates multiple molecular therapeutic targets in human leukemia. Blood 112, 1382–1391 10.1182/blood-2008-02-138958 18511810 PMC2515133

[14] Chen L., Luo L. F., Lu J., Li L., Liu Y. F., Wang J., Liu H., Song H., Jiang H., Chen S. J., Luo C., and Li K. K. (2014) FTY720 induces apoptosis of M2 subtype acute myeloid leukemia cells by targeting sphingolipid metabolism and increasing endogenous ceramide levels. PloS One 9, e103033 10.1371/journal.pone.0103033 25050888 PMC4106898

[15] Roberts K. G., Smith A. M., McDougall F., Carpenter H., Horan M., Neviani P., Powell J. A., Thomas D., Guthridge M. A., Perrotti D., Sim A. T., Ashman L. K., and Verrills N. M. (2010) Essential requirement for PP2A inhibition by the oncogenic receptor c-KIT suggests PP2A reactivation as a strategy to treat c-KIT+ cancers. Cancer Res. 70, 5438–5447 10.1158/0008-5472.CAN-09-2544 20551067 PMC2933456

[16] Ruvolo P. P., Ruvolo V. R., Jacamo R., Burks J. K., Zeng Z., Duvvuri S. R., Zhou L., Qiu Y., Coombes K. R., Zhang N., Yoo S. Y., Pan R., Hail N. Jr, Konopleva M., Calin G., Kornblau S. M., and Andreeff M. (2014) The protein phosphatase 2A regulatory subunit B55α is a modulator of signaling and microRNA expression in acute myeloid leukemia cells. Biochim. Biophys. Acta 1843, 1969–1977 10.1016/j.bbamcr.2014.05.006 24858343 PMC4165504

[17] Yang Y., Huang Q., Lu Y., Li X., and Huang S. (2012) Reactivating PP2A by FTY720 as a novel therapy for AML with C-KIT tyrosine kinase domain mutation. J. Cell. Biochem. 113, 1314–1322 10.1002/jcb.24003 22109829

[18] Shaul Y. D., and Seger R. (2007) The MEK/ERK cascade: from signaling specificity to diverse functions. Biochim. Biophys. Acta 1773, 1213–1226 10.1016/j.bbamcr.2006.10.005 17112607

[19] McCubrey J. A., Steelman L. S., Chappell W. H., Abrams S. L., Wong E. W., Chang F., Lehmann B., Terrian D. M., Milella M., Tafuri A., Stivala F., Libra M., Basecke J., Evangelisti C., Martelli A. M., and Franklin R. A. (2007) Roles of the Raf/MEK/ERK pathway in cell growth, malignant transformation and drug resistance. Biochim. Biophys. Acta 1773, 1263–1284 10.1016/j.bbamcr.2006.10.001 17126425 PMC2696318

[20] Saied M. H., Marzec J., Khalid S., Smith P., Down T. A., Rakyan V. K., Molloy G., Raghavan M., Debernardi S., and Young B. D. (2012) Genome wide analysis of acute myeloid leukemia reveal leukemia specific methylome and subtype specific hypomethylation of repeats. PloS One 7, e33213 10.1371/journal.pone.0033213 22479372 PMC3315563

[21] Lunghi P., Tabilio A., Pinelli S., Valmadre G., Ridolo E., Albertini R., Carlo-Stella C., Dall'Aglio P. P., Pelicci P. G., and Bonati A. (2001) Expression and activation of SHC/MAP kinase pathway in primary acute myeloid leukemia blasts. Hematol. J. 2, 70–80 10.1038/sj.thj.6200095 11423998

[22] Lunghi P., Tabilio A., Dall'Aglio P. P., Ridolo E., Carlo-Stella C., Pelicci P. G., and Bonati A. (2003) Downmodulation of ERK activity inhibits the proliferation and induces the apoptosis of primary acute myelogenous leukemia blasts. Leukemia 17, 1783–1793 10.1038/sj.leu.2403032 12970778

[23] Lee T. C., Ou M. C., Shinozaki K., Malone B., and Snyder F. (1996) Biosynthesis of *N*-acetylsphingosine by platelet-activating factor: sphingosine CoA-independent transacetylase in HL-60 cells. J. Biol. Chem. 271, 209–217 10.1074/jbc.271.1.209 8550561

[24] Levy M., and Futerman A. H. (2010) Mammalian ceramide synthases. IUBMB Life 62, 347–356 20222015 10.1002/iub.319 PMC2858252

[25] Sobue S., Iwasaki T., Sugisaki C., Nagata K., Kikuchi R., Murakami M., Takagi A., Kojima T., Banno Y., Akao Y., Nozawa Y., Kannagi R., Suzuki M., Abe A., Naoe T., and Murate T. (2006) Quantitative RT-PCR analysis of sphingolipid metabolic enzymes in acute leukemia and myelodysplastic syndromes. Leukemia 20, 2042–2046 10.1038/sj.leu.2404386 16990773

[26] Pitson S. M., Xia P., Leclercq T. M., Moretti P. A., Zebol J. R., Lynn H. E., Wattenberg B. W., and Vadas M. A. (2005) Phosphorylation-dependent translocation of sphingosine kinase to the plasma membrane drives its oncogenic signalling. J. Exp. Med. 201, 49–54 10.1084/jem.20040559 15623571 PMC2212769

[27] Falini B., Mecucci C., Tiacci E., Alcalay M., Rosati R., Pasqualucci L., La Starza R., Diverio D., Colombo E., Santucci A., Bigerna B., Pacini R., Pucciarini A., Liso A., Vignetti M., et al (2005) Cytoplasmic nucleophosmin in acute myelogenous leukemia with a normal karyotype. New Engl. J. Med. 352, 254–266 10.1056/NEJMoa041974 15659725

[28] Kitatani K., Akiba S., Hayama M., and Sato T. (2001) Ceramide accelerates dephosphorylation of extracellular signal-regulated kinase 1/2 to decrease prostaglandin D_2_ production in RBL-2H3 cells. Arch. Biochem. Biophys. 395, 208–214 10.1006/abbi.2001.2573 11697858

[29] Burgess M. R., Hwang E., Firestone A. J., Huang T., Xu J., Zuber J., Bohin N., Wen T., Kogan S. C., Haigis K. M., Sampath D., Lowe S., Shannon K., and Li Q. (2014) Preclinical efficacy of MEK inhibition in Nras-mutant AML. Blood 124, 3947–3955 10.1182/blood-2014-05-574582 25361812 PMC4271180

[30] Zhang W., Konopleva M., Burks J. K., Dywer K. C., Schober W. D., Yang J. Y., McQueen T. J., Hung M. C., and Andreeff M. (2010) Blockade of mitogen-activated protein kinase/extracellular signal-regulated kinase kinase and murine double minute synergistically induces Apoptosis in acute myeloid leukemia via BH3-only proteins Puma and Bim. Cancer Res. 70, 2424–2434 10.1158/0008-5472.CAN-09-0878 20215498 PMC2840060

[31] Kim W. J., Okimoto R. A., Purton L. E., Goodwin M., Haserlat S. M., Dayyani F., Sweetser D. A., McClatchey A. I., Bernard O. A., Look A. T., Bell D. W., Scadden D. T., and Haber D. A. (2008) Mutations in the neutral sphingomyelinase gene SMPD3 implicate the ceramide pathway in human leukemias. Blood 111, 4716–4722 10.1182/blood-2007-10-113068 18299447 PMC2343601

[32] Pandher R., Ducruix C., Eccles S. A., and Raynaud F. I. (2009) Cross-platform Q-TOF validation of global exo-metabolomic analysis: application to human glioblastoma cells treated with the standard PI 3-Kinase inhibitor LY294002. J. Chromatogr. B Anal. Technol. Biomed. Life Sci. 877, 1352–1358 10.1016/j.jchromb.2008.12.001 19101213

[33] Shaner R. L., Allegood J. C., Park H., Wang E., Kelly S., Haynes C. A., Sullards M. C., and Merrill A. H. Jr. (2009) Quantitative analysis of sphingolipids for lipidomics using triple quadrupole and quadrupole linear ion trap mass spectrometers. J. Lipid Res. 50, 1692–1707 10.1194/jlr.D800051-JLR200 19036716 PMC2724058

[34] Qin J., Berdyshev E., Goya J., Natarajan V., and Dawson G. (2010) Neurons and oligodendrocytes recycle sphingosine 1-phosphate to ceramide: significance for apoptosis and multiple sclerosis. J. Biol. Chem. 285, 14134–14143 10.1074/jbc.M109.076810 20215115 PMC2863199

[35] Spassieva S., Bielawski J., Anelli V., and Obeid L. M. (2007) Combination of C(17) sphingoid base homologues and mass spectrometry analysis as a new approach to study sphingolipid metabolism. Methods Enzymol. 434, 233–241 10.1016/S0076-6879(07)34012-3 17954250

[36] Pluskal T., Castillo S., Villar-Briones A., and Orešič M. (2010) MZmine 2: modular framework for processing, visualizing, and analyzing mass spectrometry-based molecular profile data. BMC Bioinformatics 11, 395 10.1186/1471-2105-11-395 20650010 PMC2918584

