# Supplementary material for: Repression of sphingosine kinase (SK)-interacting protein (SKIP) in acute myeloid leukemia diminishes SK activity and its re-expression restores SK function
Source: J Biol Chem. 2020 Mar 11;295(16):5496–508. doi: 10.1074/jbc.RA119.010467 (PMC7170527; doi:10.1074/jbc.RA119.010467)
Supplement: Supporting Information [file supp_295_16_5496__index.html]

Repression of sphingosine kinase (SK)-interacting protein (SKIP) in acute myeloid leukemia diminishes SK activity and its re-expression restores SK function — SKIP enhances sphingosine kinase function — Repression of sphingosine kinase (SK)-interacting protein (SKIP) in acute myeloid leukemia diminishes SK activity and its re-expression restores SK function — SKIP enhances sphingosine kinase function — Supporting Information 

# Repression of sphingosine kinase (SK)-interacting protein (SKIP) in acute myeloid leukemia diminishes SK activity and its re-expression restores SK function

## Supporting Information

- Supporting Information (to be published online) - Table S1-2 Figures S1-9
