# Supplementary material for: Repression of sphingosine kinase (SK)-interacting protein (SKIP) in acute myeloid leukemia diminishes SK activity and its re-expression restores SK function
Source: J Biol Chem. 2020 Mar 11;295(16):5496–508. doi: 10.1074/jbc.RA119.010467 (PMC7170527; doi:10.1074/jbc.RA119.010467)
Supplement: Supporting Information [file supp_RA119.010467_154926_2_supp_487004_q6hk54.pdf]

Supporting information

Repression of sphingosine kinase (SK)-interacting protein (SKIP) in acute myeloid leukemia diminishes SK activity and its re-expression restores SK function

Essam A Ghazaly[1]*, Farideh Miraki-Moud[1,2], Paul Smith[1], Chathunissa Gnanaranjan[1], Lola Koniali[1], Adedayo Oke[1], Marwa H Saied[1], Robert Petty[1], Janet Matthews[1], Randal Stronge[2,3], Simon P. Joel[1], Bryan D. Young[1], John Gribben[1] and David C. Taussig[1,2,3,*].

Running title: SKIP enhances sphingosine kinase function

[1]Centre for Haemato-Oncology, Barts Cancer Institute, Queen Mary University of London, London EC1M 6BQ, UK; [2]Institute of Cancer Research, Sutton, London; [3]Department of Haematology, Royal Marsden Hospital, Sutton, United Kingdom

* Correspondence to David C Taussig: Haemato-Oncology, Royal Marsden Hospital, Downs Road, Sutton, Surrey, SM2 5PT, UK; david.taussig@icr.ac.uk; Telephone +44 208 661 3655; Fax: +44 208 642 9634; and Essam A Ghazaly: Pharmacokinetics group, Medicines and Healthcare products Regulatory Agency (MHRA), London E14 4PU, UK; e.a.ghazaly@qmul.ac.uk

Table S1: Primary AML cell samples characteristics (MRC, Medical Research Council). * = Leucopheresis

AML Sample Number	Cytogenetics	MRC Cytogenetic risk group	WBC at diagnosis (10^9/L)	% Blast Cells	Sample type
1	t(8;21)	Good	73	95	PB*
2	t(8;21)	Good	7.6	1	PB
3	t(8;21)	Good	17.9	74	PB
4	t(8;21)	Good	197	20	PB
5	t(8;21)	Good	24.7	39	PB
6	t(8;21)	Good	70	79	PB
7	inv (16)	Good	42.6	56	PB*
8	inv (16)	Good	6.3	22	PB*
9	inv (16)	Good	30	88	PB
10	inv (16) +	Good	22.6	3	PB
11	t(15;17)	Good	46.9	85	PB
12	t(15;17)	Good	9.4	64	PB
13	t(15;17)	Good	29.5	70	PB*
14	t(15;17)	Good	202.5	96	PB*
15	Normal	Intermediate	120.1	70	PB*
16	Normal	Intermediate	89.5	89	PB
17	Normal	Intermediate	105.1	97	PB*
18	Normal	Intermediate	4.6	2	PB*
19	Normal	Intermediate	29.3	55	PB
20	Normal	Intermediate	100	74	PB
21	Normal	Intermediate	85.3	83	PB
22	Normal	Intermediate	32.8	61	PB*
23	+8, DER(12),t(3;12)involving EVI(3Q26)	Poor	37	35	PB
24	Complex	Poor	3.4	10	PB*
25	Isochromosome 22q	Intermediate	76.7	44	BM
26	Normal	Intermediate	30	90	BM
27	17p-	Poor	26	90	BM
30	7q-	Poor	34.5	61	BM

Table S2: AML plasma samples characteristics (MRC, Medical Research Council)

AML Sample Number	Cytogenetics	MRC Cytogenetic risk group
1	t(8;21)	Good
2	t(8;21)	Good
3	Normal	Intermediate
4	Normal	Intermediate
5	Normal	Intermediate
6	Normal	Intermediate
7	Normal	Intermediate
8	Normal	Intermediate
9	Trisomy 6, Trisomy 8	Intermediate
10	Trisomy 11	Intermediate
11	del9q	Intermediate
12	del 5q	Poor
13	t(2;9;22)	Poor
14	Complex	Poor
15	Complex	Poor

Figure S1
SK1 and SK2 expression in primary leukemia cells.

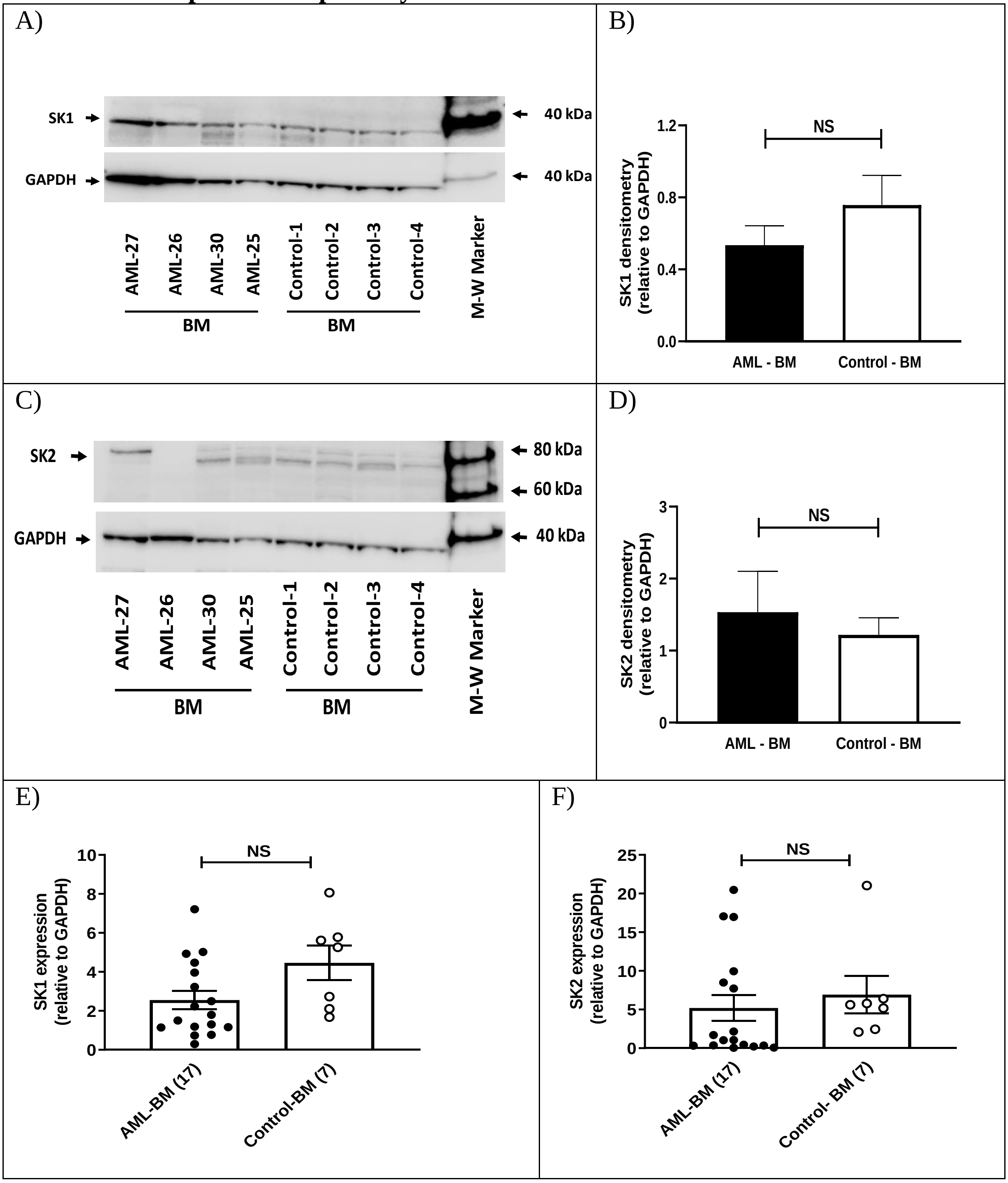

Figure S1

SK1 and SK2 expression in primary leukemia cells.
Western blots and densitometry normalised to GAPDH show SK1 (**A** and **B**) and SK2 (**C** and **D**) expression in bone marrow from AML patients and control subjects. Quantitative real time PCR data analysis of SK1 (**E**) and SK2 (**F**) in bone marrow from AML patients and control

subjects. There was no significant difference between the expression of SK1 or SK2 comparing primary AML cells to controls.

Figure S2
SKIP expression is increased in SKIP transfected cell lines.
A)

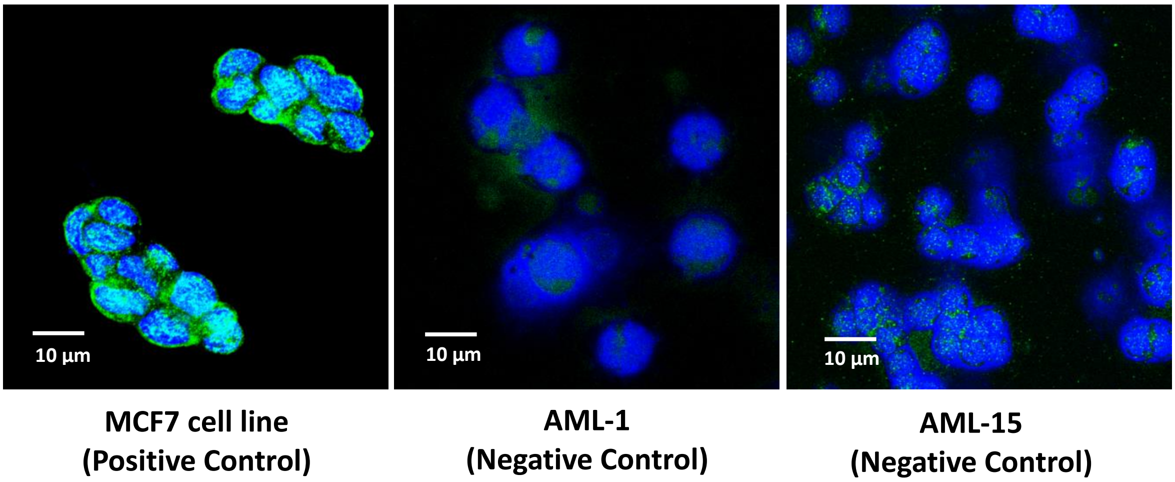

B) SKIP staining in transfected cell lines.

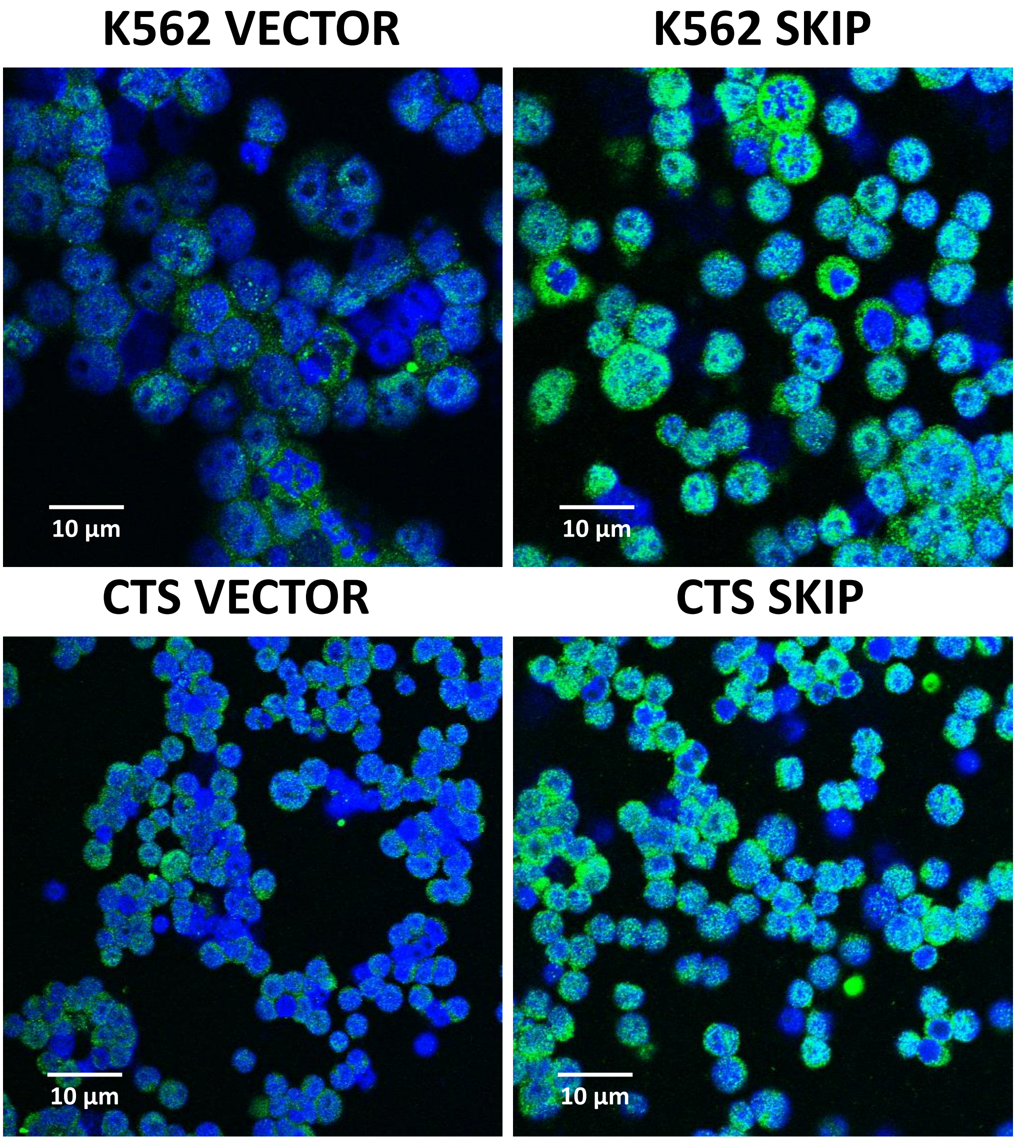

Figure S2
SKIP expression is increased in SKIP transfected cell lines.

A) MCF7 cells were used as a positive control and B) highly SKIP hypermethylated AML samples (with > 45% SPHKAP hypermethylation) as negative controls. C) SKIP expression is higher in SKIP-transfected cell lines. There is higher expression in both nucleus and cytoplasm.

Figure S3
SK1 and SK2 expression in SKIP transfected cell lines.

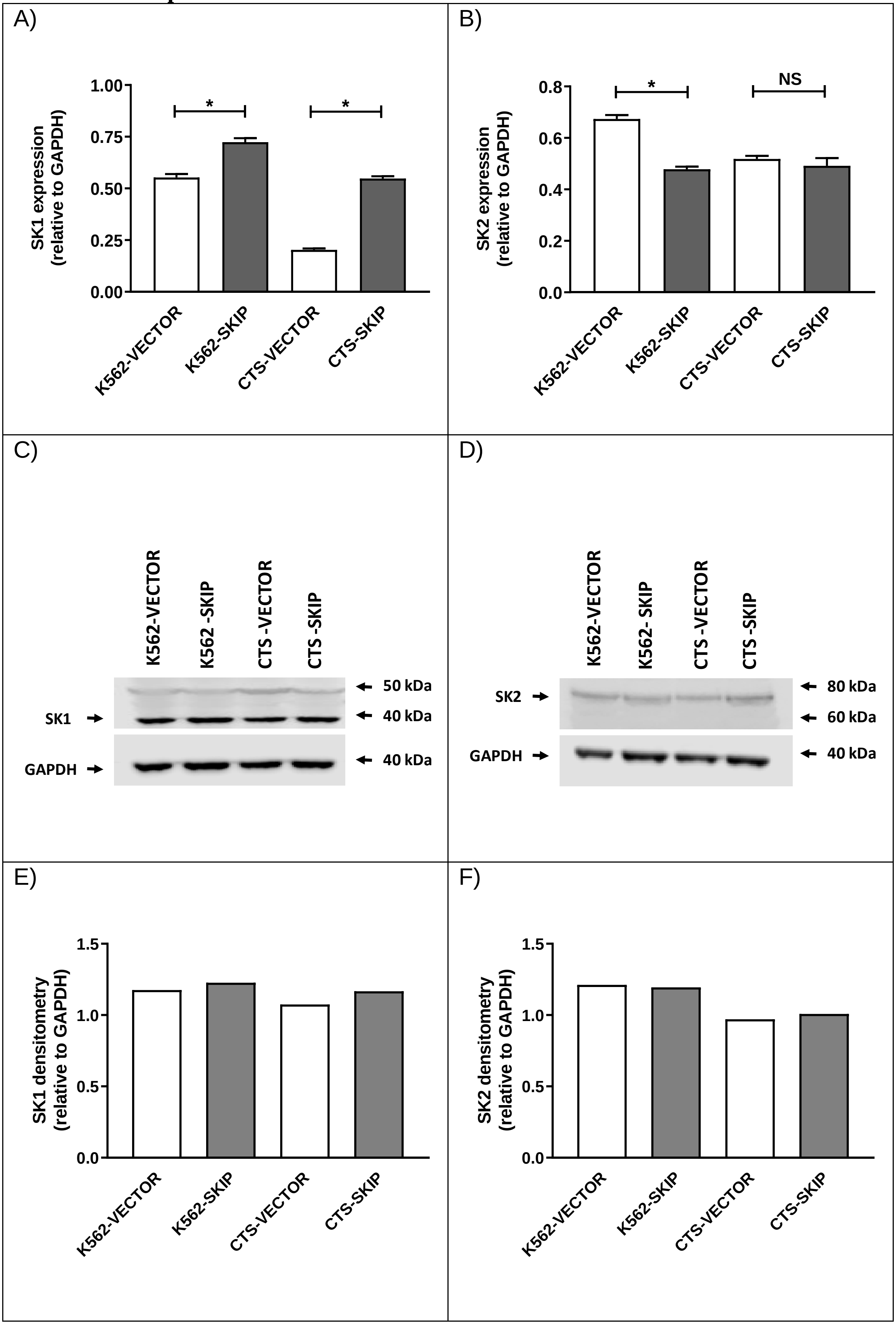

Figure S3

SK1 and SK2 expression in SKIP transfected cell lines.
Quantitative real time PCR data analysis of SK1 (**A**) and SK2 (**B**) in vector and SKIP transfected CTS and K562 cell lines. Western blots and densitometry normalised to GAPDH show equal SK1 (**C** and **E**) and SK2 (**D** and **F**) in vector and SKIP transfected CTS and K562 cell lines. There was significantly more SK1 mRNA in SKIP transfected cell lines but this did not translate into higher protein levels. SK2 mRNA was singificantly lower in SKIP transfected K562 cells but not CTS cells but again there was no difference in the protein levels.

Figure S4
Distribution of SK1 and SK2 in SKIP transfected cells

A) SK1 expression by mRNA in control cell lines

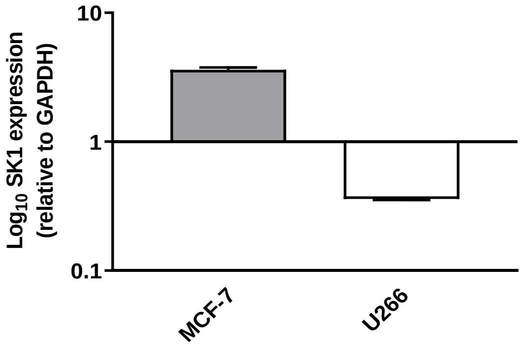

B) SK1 expression by Western blotting in control cell lines

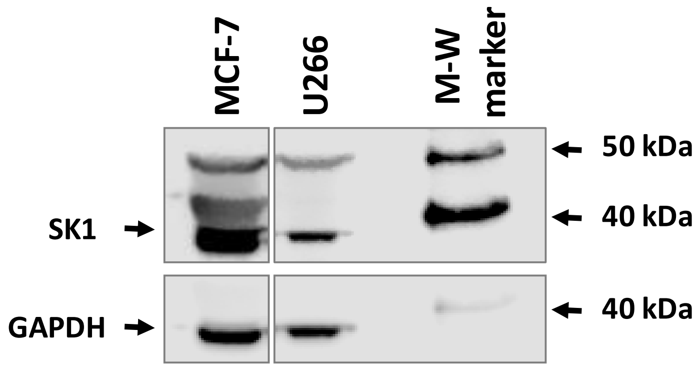

C) SK1 staining (positive and negative controls)

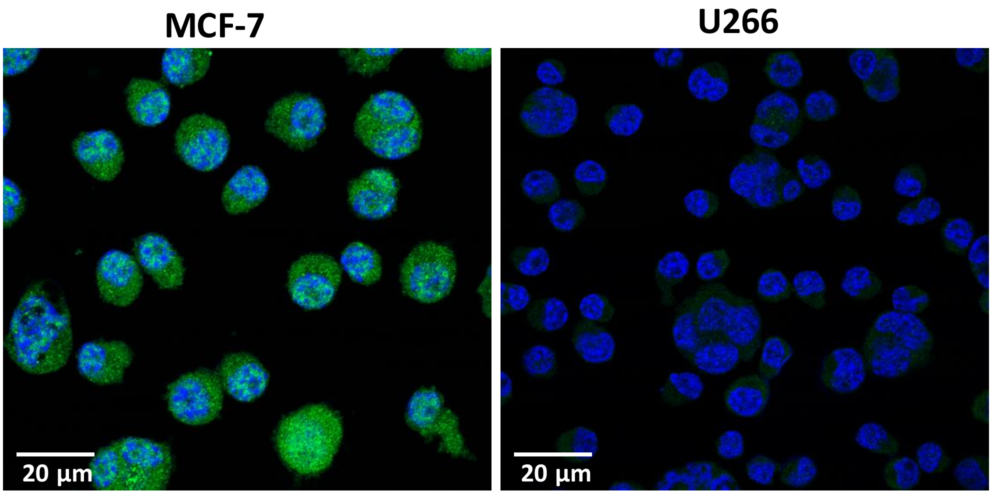

Figure S4 continued.

D) SK1 staining in SKIP transfected cells

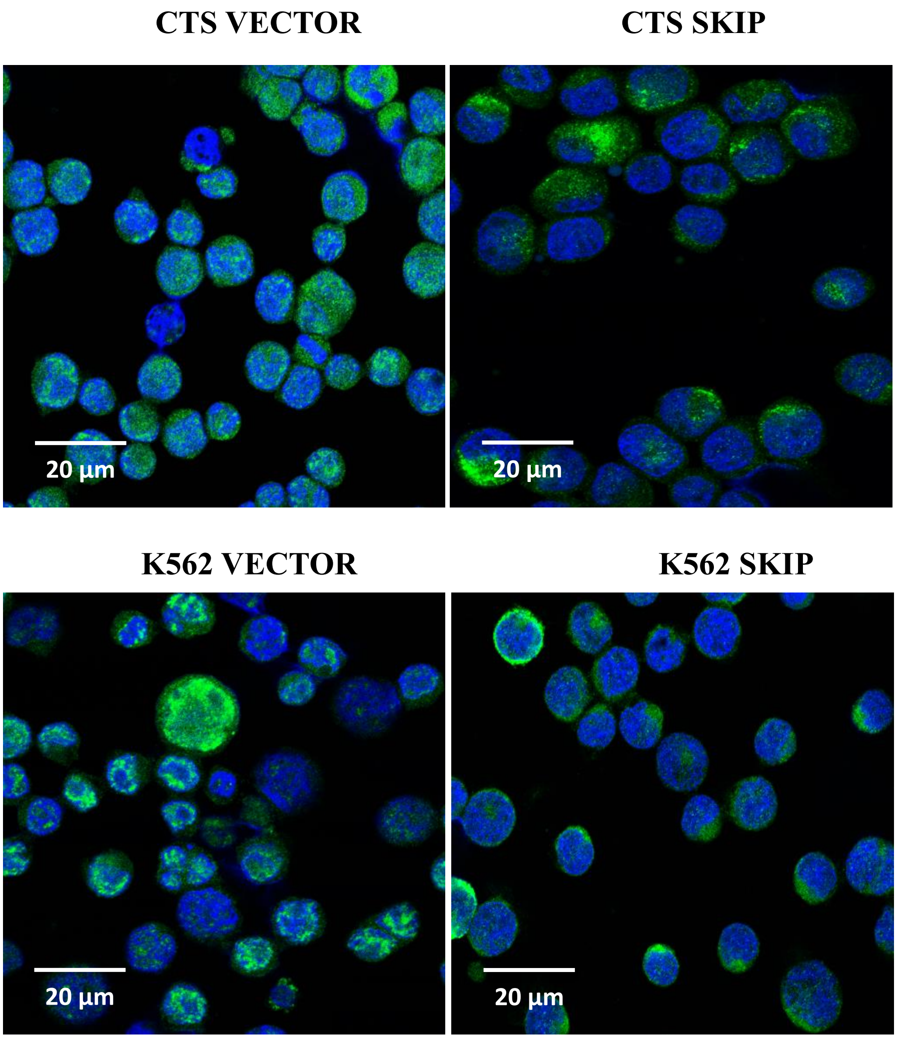

Figure S4 continued

E) SK2 stainingin SKIP transfected cells

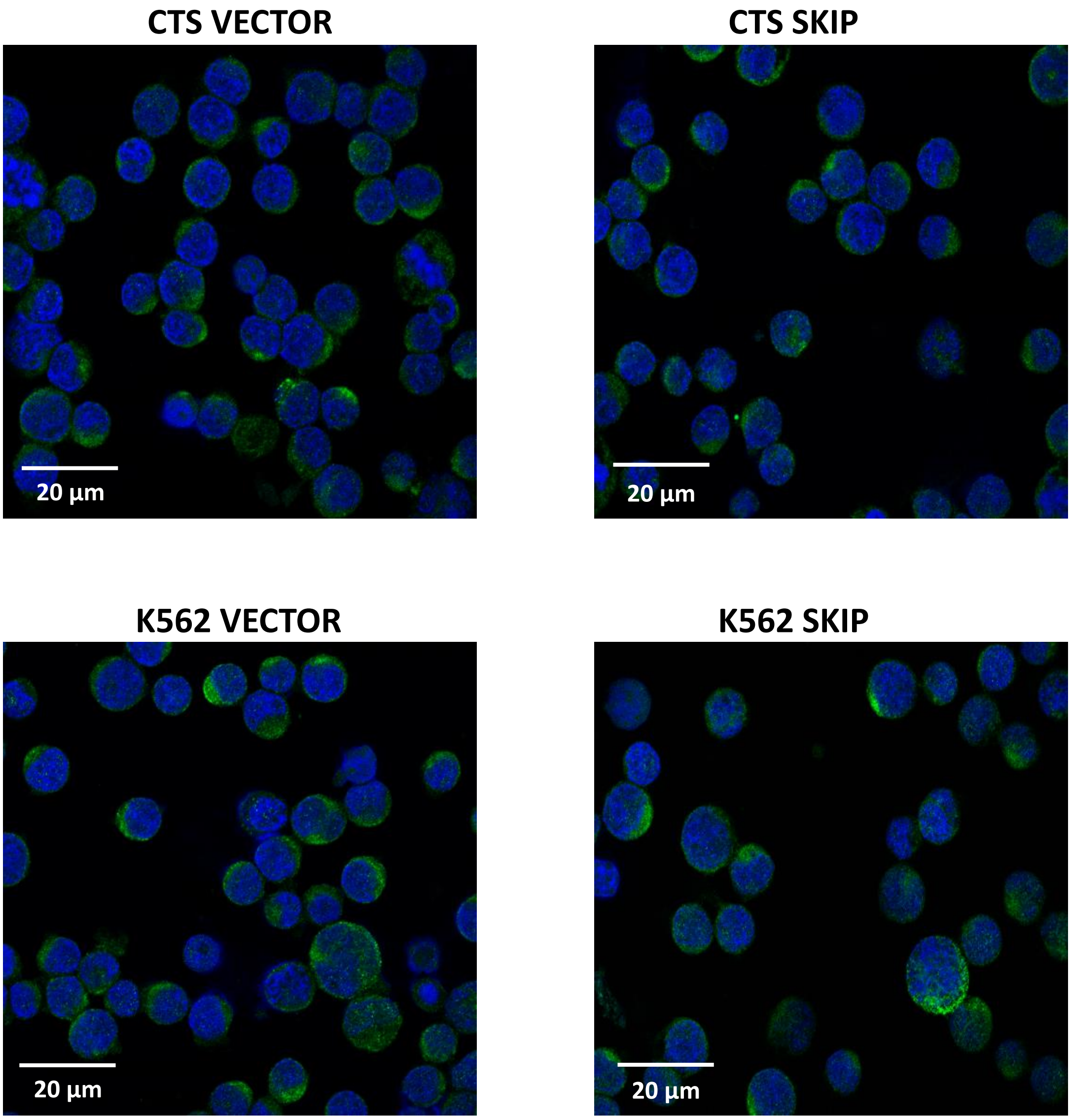

Figure S4

Distribution of SK1 and SK2 in SKIP transfected cells

Validation of anti-SK1 antibody; (**A**) quantitative real time PCR (**B**) Western blot and (**C**) immunofluorescence staining for SK1 antibody using MCF-7 breast cancer cell line as positive control and U266 myeloma cell line as negative control. (**D**) SK1 staining in CTS and K562 cell lines transfected with SKIP; the SK1 is concentrated in the cytoplasm in SKIP-transfected but is distributed across both cytoplasm and nucleus in VECTOR-transfected cells.(**E**) SK2 staining in CTS and K562 cell lines transfected with SKIP; there is no difference in the distribution of SK2 in the SKIP transfected cell lines. In both VECTOR and SKIP transfected cells distribution of SK2 is in the cytoplasm.

Figure S5
SKIP protein localises SK1 to the cell cytoplasm.

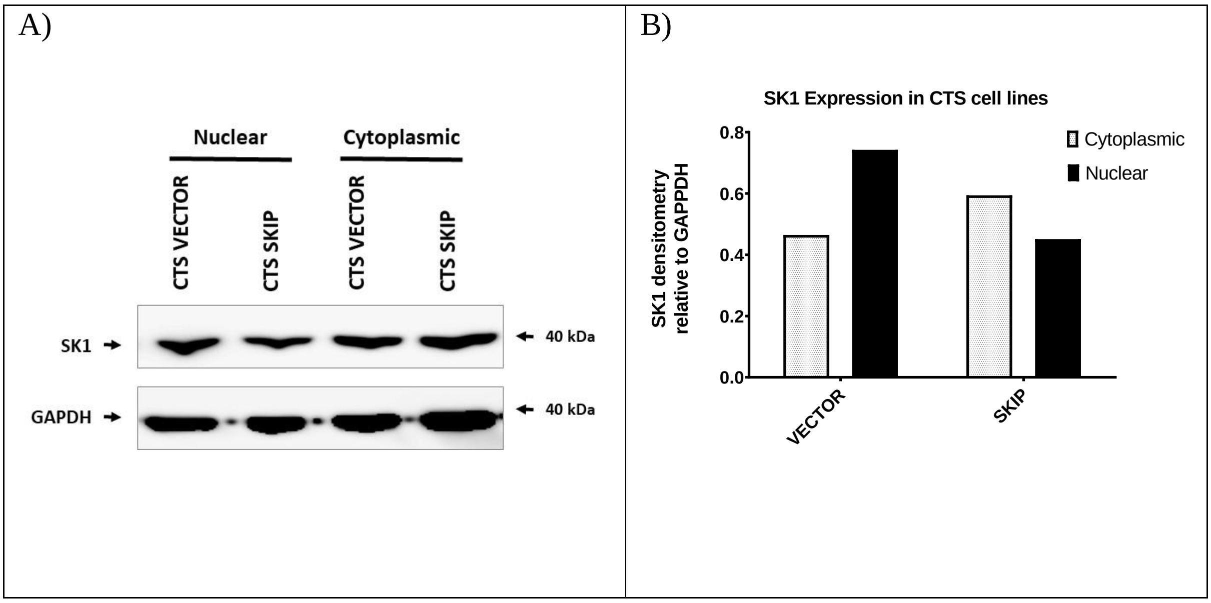

Figure S5

SKIP protein localises SK1 to the cell cytoplasm.

SK1 shows localised expression to cell cytoplasm in SKIP-transfected CTS versus nuclear expression in vector alone-transfected leukemia cell lines as assessed by Western Blot (**A**) and densitometry of SK1 expression normalised to GAPDH (**B**).

Figure S6
Untargeted metabolomic changes associated with SKIP gene transfection inside and outside K562 leukemic cells

A) K562 intracellular metabolite PCA

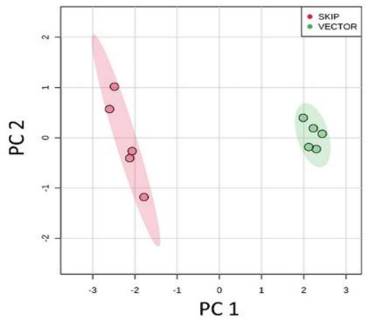

B) K562 intracellular metabolite heatmap

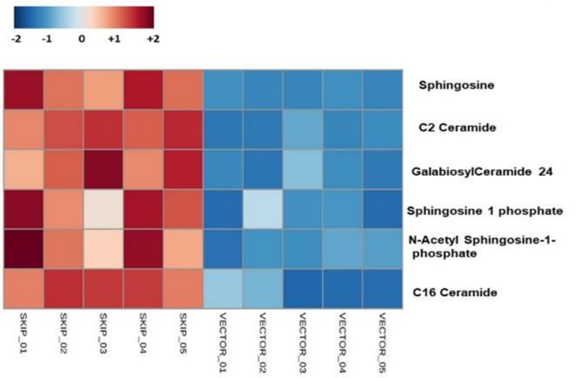

C) K562 Extracellular metabolite PCA

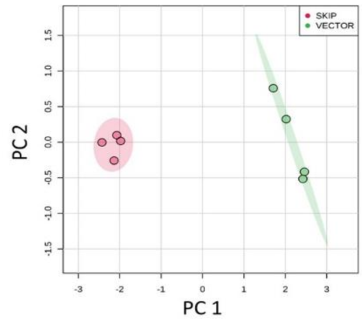

D) K562 Extracellular metabolite Heatmap

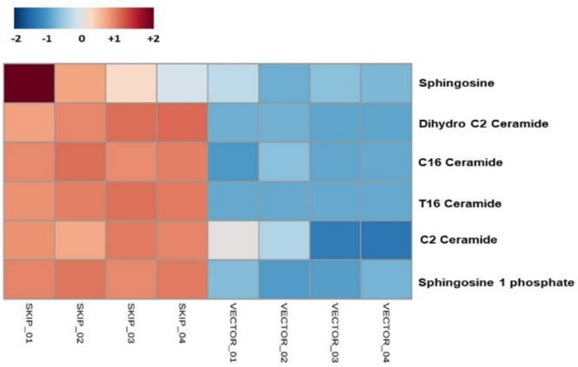

E) CTS intracellular metabolite PCA

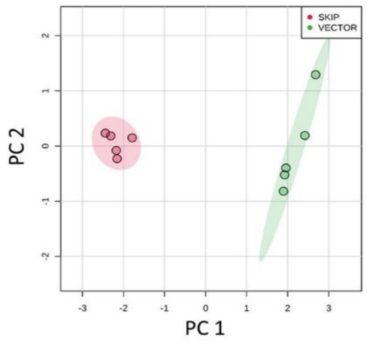

F) CTS intracellular metabolite heatmap

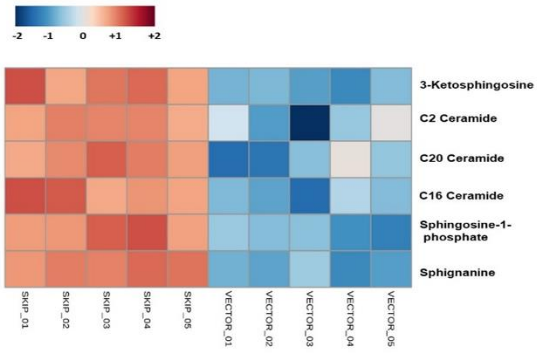

G) Primary cells metabolite PCA

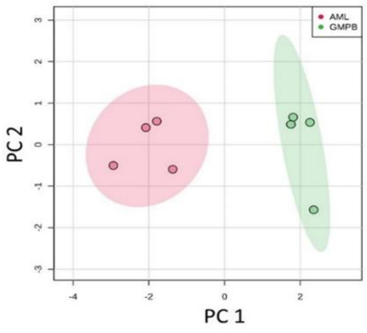

H) Primary cells metabolite heatmap

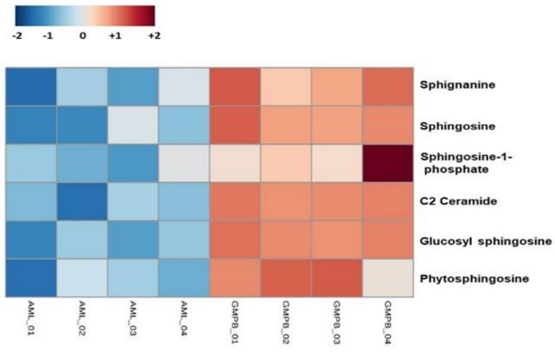

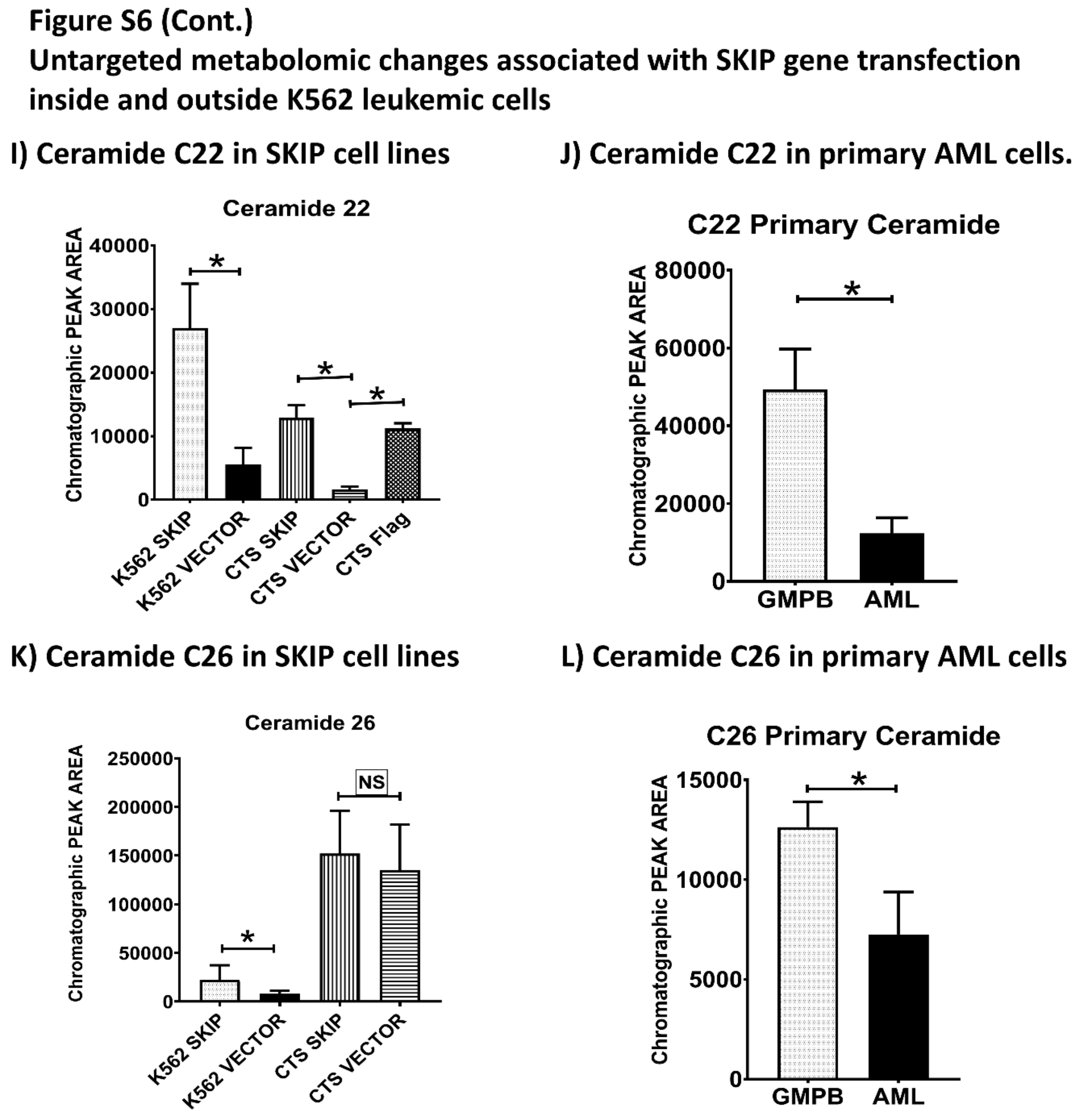

Figure S6

Untargeted metabolomic changes associated with SKIP gene transfection inside and outside K562 leukemic cells.

The intracellular metabolome was assayed using untargeted UPLC-MS metabolomic analysis as described in the methods section under the title of Untargeted UPLC-MS metabolomic analysis. The identified metabolites and their peak areas were then analysed using MetaboAnalyst online tool (https://www.metaboanalyst.ca/) where principle component analyses (PCA), plotting the first two principle components (PC1 versus PC2), showed clear

separation between sphingosine kinase interacting protein (SKIP) and vector alone (VEC) transfected K562 cells **(A)** and the Heatmap for the top metabolic changes all involved sphingolipid metabolites (**B**). Extracellular cellular metabolites were also assayed using untargeted UPLC-MS metabolomic analysis of culture medium incubated with K562 cell lines for 72 hrs and PCA **(C)** and Heatmaps **(D)** are shown. Similar observations were obtained from CTS cell lines (PCA and Heatmaps are shown in panels **(E)** and **(F),** respectively) and AML primary cell lines compared to GMPB cells (PCA and Heatmaps are shown in panels **(G)** and **(H),** respectively). Untargeted UPLC-MS analysis also detected changes in ceramides C22 and C26. Data for C22 ceramide from cell lines is shown in panel **(I)** and data from primary AML cells is shown in panel **(J)**. Data for ceramide C26 from cell lines is shown in panel **(K)** and data from primary AML cells is shown in panel **(L)**). Asterisk signifies statistical significance by unpaired T test (P <0.05).

Figure S7

Effect of SKIP transfection on chemosensitivity.

Figure S7

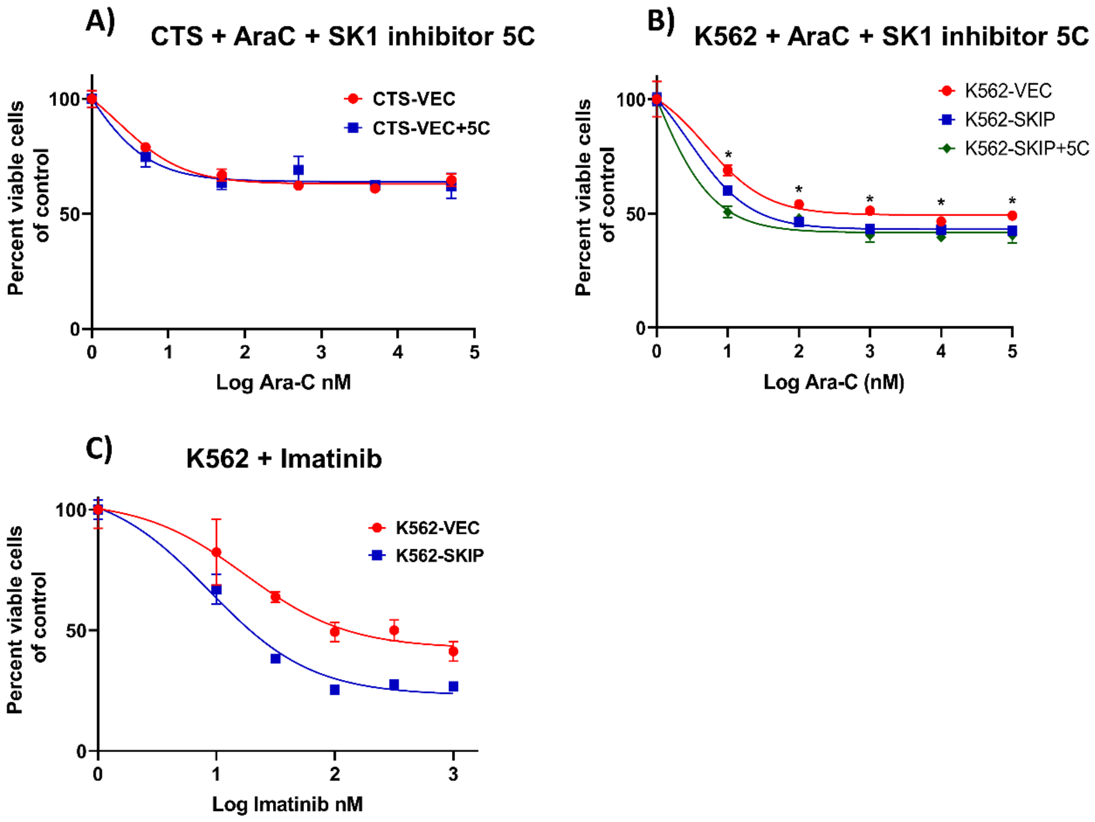

Figure S7

Effect of SKIP transfection on chemosensitivity.

The SK1 inhibitor 5C did not affect the response to ara-c chemotherapy in CTS vector-transfected cells (A). SKIP-transfected K562 cells were more sensitive to ara-c Chemotherapy than vector transfected cells (B). The asterisks indicate statistical significance using a T Test comparing SKIP and VEC transfected cells at each dose point ($p < 0.05$)(n=3). The SK1 inhibitor 5C did not reverse the pro-apoptotic effect of SKIP-transfection. (C) SKIP-transfected K562 leukemia cell lines were more sensitive to imatinib after a 72 hour incubation period (n=3).

Figure S8

Validation of anti-SK2 antibody

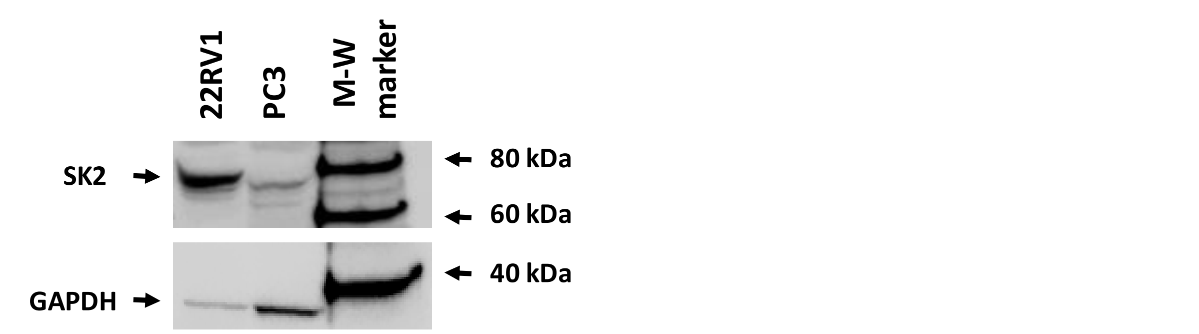

Figure S8

Validation of anti-SK2 antibody

Validation of anti-SK2 antibody, using 22RV1 and PC3 cell lines (prostate cancer cell lines) as positive controls.

Figure S9

Targeted UPLC-MS/MS analysis

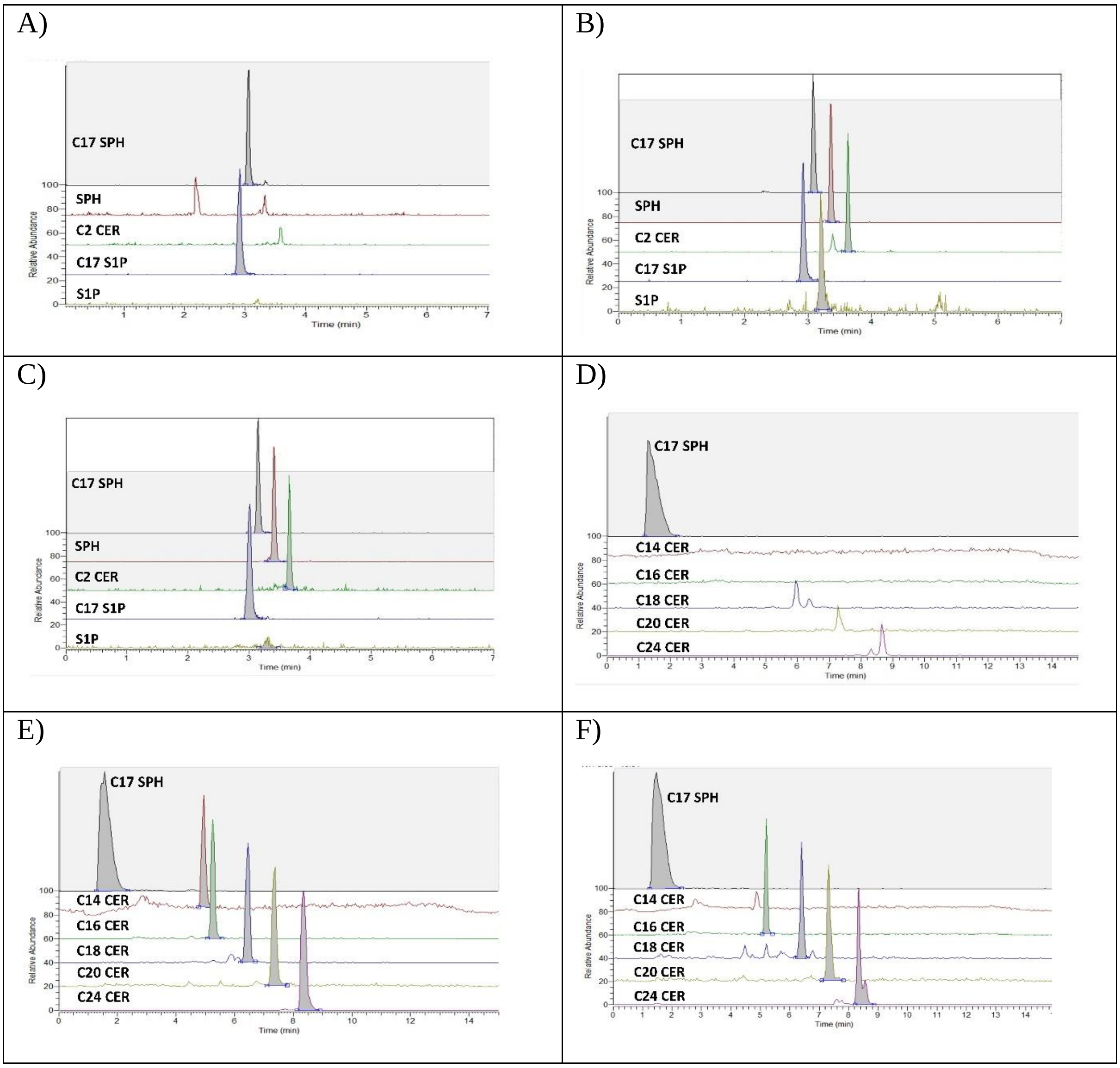

Figure S9

Targeted UPLC-MS/MS analysis

Representative chromatograms for SPH, S1P and C2 ceramide LC-MS/MS assay: (**A**) blank (negative control) sample spiked before extraction with internal standards C17 sphingosine (SPH) and C17 sphingosine 1 phosphate (S1P), (**B**) extracted control sample spiked with 1 μM of SPH, S1P and C2 ceramide before extraction, (**C**) extracted GMPB sample.

Representative chromatograms for long chain ceramide assay: (**D**) blank (negative control) sample spiked before extraction with internal standards C17 sphingosine (SPH), (**E**) extracted control sample spiked with 1 μM of long chain ceramide mix, (**F**) extracted GMPB sample.
